# New Antiproliferative Cembrane Diterpenes from the Red Sea *Sarcophyton* Species

**DOI:** 10.3390/md17070411

**Published:** 2019-07-11

**Authors:** Hossam M. Hassan, Mostafa E. Rateb, Marwa H. Hassan, Ahmed M. Sayed, Samah Shabana, Mai Raslan, Elham Amin, Fathy A. Behery, Osama M. Ahmed, Abdullatif Bin Muhsinah, Tobias A. M. Gulder, Usama Ramadan Abdelmohsen

**Affiliations:** 1 Department of Pharmacognosy, Faculty of Pharmacy, Beni-Suef University, Beni-Suef 62514, Egypt; 2 School of Computing, Engineering & Physical Sciences, University of the West of Scotland, Paisley PA1 2BE, UK; 3 Department of Pharmacognosy, Faculty of Pharmacy, Nahda University, Beni-Suef 62514, Egypt; 4 Pharmacognosy Department, College of Pharmaceutical Sciences & Drug Manufacturing, Misr University for Science and Technology (MUST), Six October City 77, Egypt; 5 Biotechnology and Life Sciences Department, Faculty of Postgraduate Studies for Advanced Sciences, Beni-Suef University, Beni-Suef 62511, Egypt; 6 Department of Pharmacognosy, Faculty of Pharmacy, Mansoura University, Mansoura 35516, Egypt; 7 Department of Pharmaceutical Sciences, College of Pharmacy, Riyadh Elm University, Riyadh 11681, Saudi Arabia; 8 Physiology Division, Zoology Department, Faculty of Science, Beni-Suef University, Beni-Suef 62514, Egypt; 9 Department of Pharmacognosy, College of Pharmacy, King Khalid University, Abha 61441, Saudi Arabia; 10 Chair of Technical Biochemistry, Department of Chemistry and Food Chemsitry, Technical University of Dresden, Bergestrabe 66, Dresden 01069, Germany; 11 Department of Pharmacognosy, Faculty of Pharmacy, Minia University, Minia 61519, Egypt

**Keywords:** LCHRESMS, dereplication, cembranoids, *Sarcophyton*, antiproliferative

## Abstract

The combination of liquid chromatography coupled to high resolution mass spectrometry (LC-HRESMS)-based dereplication and antiproliferative activity-guided fractionation was applied on the Red Sea-derived soft coral *Sarcophyton* sp. This approach facilitated the isolation of five new cembrane-type diterpenoids (**1**–**5**), along with two known analogs (**6** and **7**), as well as the identification of 19 further, known compounds. The chemical structures of the new compounds were elucidated while using comprehensive spectroscopic analyses, including one-dimensional (1D) and two-dimensional (2D) NMR and HRMS. All of the isolated cembranoids (**1**–**7**) showed moderate *in vitro* antiproliferative activity against a human breast cancer cell line (MCF-7), with IC_50_ ranging from 22.39–27.12 µg/mL. This class of compounds could thus serve as scaffold for the future design of anticancer leads.

## 1. Introduction

Marine natural products are characterized by their diversity in chemical structures, along with biological activities [1]. The Red Sea is considered to be one of the most important marine spots comprising high biodiversity. About 40% of the soft corals that are identified worldwide are native to the Red Sea, however only a few species that have been chemically examined in the last decades [1,2,3]. The genus *Sarcophyton* (Phylum Cnidaria; Order Alcyonacea; Family Alcyoniidae) contains 46 species with typically mushroom- or toadstool-shaped appearance and it is considered one of the most abundant Red Sea soft corals [1]. *Sarcophyton* sp. are well recognized as a rich source for a wide range of terpenoid metabolites. Macrocyclic cembrane-type diterpenoids and their derivatives represent the main chemical defense for *Sarcophyton* species against natural predators [3,4,5]. Previous studies concerning the biological activities of cembranoid analogues revealed that they exhibited a wide range of biological activities, including ichthyotoxic [6], anti-inflammatory [7], anti-bacterial [8], neuroprotective [9], and antitumor [10] properties. In addition, a number of *Sarcophyton*-derived cembranoid analogues (e.g., sarcophine and its hydroxylated derivatives) were investigated as potential competitive cholinesterase inhibitors [11], selective noncompetitive phosphofructokinase inhibitors [12], and a Na^+^, K^+^-ATPase inhibitors [13]. 

Cancer chemoprevention is based on chemical compounds that inhibit or reverse the development of cancer in normal or pre-neoplastic tissue [14]. A lot of novel marine metabolites were identified as anticancer leads during the past 20 years [15]. Our previous work on sarcophine (**7**) and its analogs has illustrated the ability of this class of compounds to inhibit growth, proliferation, and migration of the prostate and breast metastatic cancer cell lines PC-3 and MDA-MB-231 [6]. 

**Figure 1:**
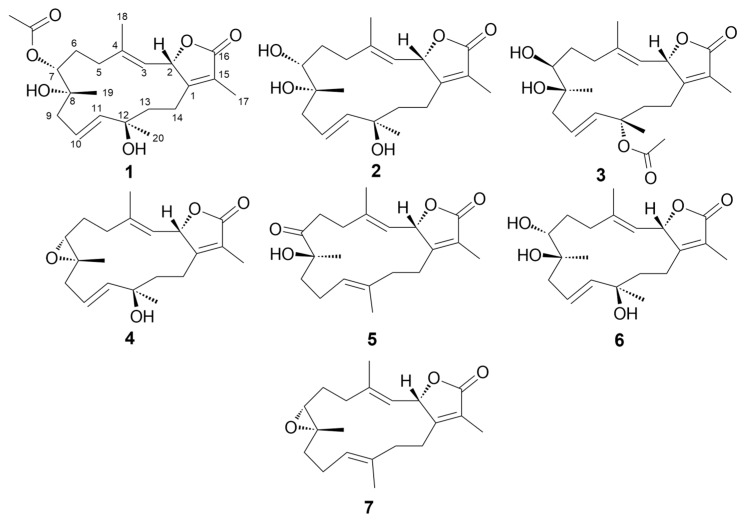
Structures of isolated compounds (**1**–**7**).

Looking for new natural products with cytotoxic activity that is applicable in cancer therapy is of utmost importance. The discovery process has improved with the evolution of new spectroscopic techniques. Liquid chromatography coupled to high resolution mass spectrometry (LC-HRESMS) can generate information-rich data sets that can assist in the dereplication of previously reported natural products that are present in the crude extracts prior to a tedious isolation attempt. New biological material was collected and extracted in order to extend our previous investigation of the Red Sea *Sarcophyton* species [6]. Subsequently, bioactivity-guided isolation assisted by LC-HRESMS metabolic profiling led to the isolation of five new cembrane-type diterpenoids (**1**–**5**, Figure 1) that are structurally related to sarcophine (**7**) which was also isolated along with another known cembranoid, sinumaximol G (**6**). Testing of all isolated compounds against the MCF-7 breast cancer cell line showed a moderate *in vitro* growth inhibitory activity. 

## 2. Results and Discussion

Extraction and fractionation on normal phase (NP) silica gel while using medium pressure liquid chromatography (MPLC) afforded seven major fractions. LC-HRESMS dereplication of these fractions using commercial and public databases led to the putative identification of 19 compounds, which were previously reported from *Sarcophyton* sp., in addition to 17 molecules with mass data showing no hits in MS databases, most of them in fractions 6 and 7 (Table S1). These LC-HRESMS findings, together with the *in vitro* cytotoxicity results against MCF-7 cell line using the acquired MPLC fractions, led to prioritization of fractions 6 and 7 for further chromatographic purification on silica gel and Sephadex LH-20 to isolate the active components. Preliminary ^1^H NMR spectroscopic analysis revealed that all the major compounds in fractions 6 and 7 had a cembranoid backbone [16,17], differing either in the degree of oxidation or the configuration of one or more chiral centers. 

### 2.1. Identification of the Isolated Compounds

**Figure 2:**
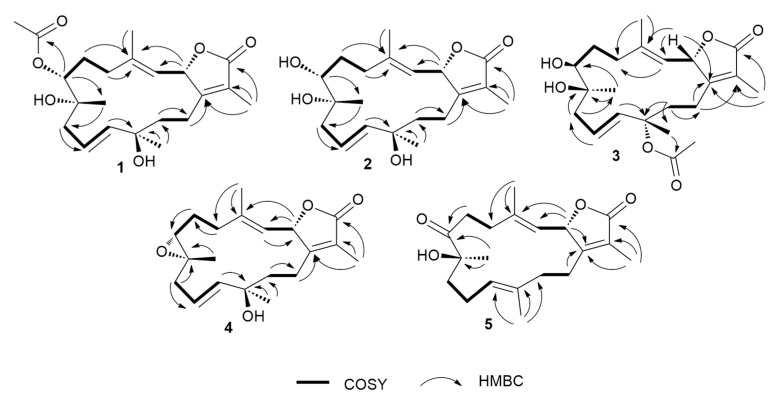
^1^H-^1^H COSY and key HMBC correlations of compounds **1**–**5**.

**Figure 3:**
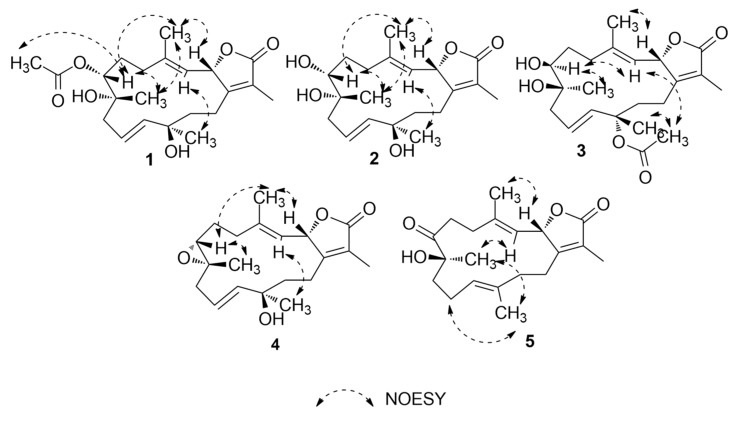
^1^H-^1^H NOESY correlations of compounds **1**–**5**.

Compound **1** was obtained as colorless oil. Its molecular formula was established as C_22_H_32_O_6_ that is based on HRESIMS data (Figure S1) that showed an [M + H]^+^ ion at *m/z* 393.2264 (calcd *m/z* 393.2272). The ^1^H NMR spectrum (Table 1 and Table 2), together with the multiplicity-edited HSQC (Figures S2–S5), were in harmony with a sarchophine (**7**) skeleton that was previously isolated from other *Sarcophyton* species [6,16,18]. ^1^H NMR and ^13^C NMR (Table 2), together with multiplicity-edited HMQC, showed the following characteristic resonances: (i) the presence of an α,β-unsaturated-γ-lactone functionality: *δ*_C_ 122.9 (C-15), *δ*c 161.4 (C-1), and *δ*c 175.4 (C-16). (ii) Two olefinic moieties at *δ*c 142.0 (C-4) and *δ*c_/H_ 122.9/4.81 (C-3), as well as at *δ*c_/H_ 124.5/ 5.48 (C-10) and *δ*_C/H_ 140.0/5.47 (C-11). HMBC correlations confirmed the positions of the olefinic moieties (Figure 2 and Figures S6 and S7) H-5/C-4, H-18/C-4, H-18/C-3, H-9/C-10, H-9/C-11, H-10/C-8, and H-10/C-12. (iii) Three oxygen functionalities; an oxomethine at *δ*_C/H_ 79.2/5.48 (C-2) and two oxygenated quaternary carbons at *δ*_C_ 74.3 (C-8) and *δ*_C_ 74.4 (C-12). (iv) Four methyl singlets at [*δ*_H_ 1.86 (H-17), *δ*_H_ 1.85 (H-18), *δ*_H_ 1.20 (H-19), and *δ*_H_ 1.36 (H-20)], and five methylene groups [*δ*_H_ 2.14, 1.90 (H-5); *δ*_H_ 1.90, 1.75 (H-6); *δ*_H_ 2.28, 2.20 (H-9); *δ*_H_ 1.70, 1.68 (H-13); and, *δ*_H_ 2.18, 2.48 (H-14)]. HMBC correlations between H_2_-14 and *δ*_C_ 161.4 (C-1), H-3 and *δ*_C_ 35.5 (C-5), H_2_-5 and *δ*_C_ 142.0 (C-4) and H2-6 and δ_C_ 35.5 (C-5) and H_2_-6 /H_2_-5 and δ_C_ 74.4 (C-7) established the carbon linkages from C-14 to C-7. Furthermore, correlations between H_2_-9 and *δ*_C_ 74.3 (C-8)/124.5 (C-10)/140.0 (C-11); H-10 and *δ*_C_ 74.3 (C-8)/74.4 (C-12); H_2_-13 and *δ*_C_ 74.4 (C-12)/140.0 (C-11)/23.0 (C-14); and finally, H_2_-14 and *δ*_C_ 161.4 (C-1)/79.2 (C-2) established the connectivity of the 14-membered ring. The positions of the methyl groups were recognized through HMBC correlations between *δ*_H_ 1.85 (H_3_-18, s) and C-3 and C-4; *δ*_H_ 1.20 (H_3_-19, s) and C-9; *δ*_H_ 1.36 (H_3_-20, s) and C-11 and C-12; and, *δ*_H_ 1.86 (H_3_-17, s) and C-1, C-15, and C-16. These NMR spectroscopic features were very similar to the reported spectroscopic data of the known compound sinumaximol G, which was previously isolated from the Soft Coral *Sinularia maxima* [16] and also isolated in the present work (Compound **6**). The ^1^H NMR and ^13^C NMR spectroscopic data of compound **1** showed differences in the chemical shifts of C-7/*δ*_C_ 74.4 and H-7/*δ*_H_ 4.82, which indicated changes of the substitution at C-7. In addition, the presence of additional resonances in the NMR spectral data for acetate moiety [*δ*_C_ 170.3 (C-1’), *δ*_C/H_ 21.1/2.08 (C-2’)], along with the HMBC correlations of H_3_-2’/C-1’, H-7/C-1’, and H-7/C-19, suggested that the OH group in sinumaximol G at C-7 has been acetylated in compound **1**. The relative configuration of **1** was determined on the basis of coupling constants and NOESY experiments (Figures S8 and S9). The vicinal coupling constant of 14.0 Hz between H-2 (*δ*_H_ 5.48) and H-3 (*δ*_H_ 4.81), together with the ^1^H-^1^H NOESY correlations (Figure 3) between the methyl protons H_3_-18 (*δ*_H_ 1.85), and both H-2 (*δ*_H_ 5.48) and H-7 (*δ*_H_ 4.82), suggested a *β*-orientation of both H-2 and H-7. Moreover, the ^1^H-^1^H NOESY correlations between H-3 (*δ*_H_ 4.81) and H-20 (*δ*_H_ 1.36) suggested that H_3_-20 were in the *α*-orientation. Alterations in the ^1^H NMR chemical shift of H_3_-19 (*δ*_H_ 1.2), together with the ^1^H-^1^H NOESY correlations between H_3_-19 (*δ*_H_ 1.2) and H_3_-18 (*δ*_H_ 1.85), indicated changes in the orientation of the methyl group at C-8 to be in the *β*-orientation. Therefore, compound **1** was assigned as a new natural product and given the name 7-acetyl-8-*epi*- sinumaximol G (**1**).

The molecular formula of compound 2 was determined to be the same as that of sinumaximol G (**6**) (C_20_H_30_O_5_); *m/z* 351.2160 [M + H]^+^ ion (calcd *m/z* 351.2166) (Figure S10). The one-dimensional (1D) and two-dimensional (2D) NMR spectroscopic features (Table 1 and Table 2; Figures S11–S15) also indicated that 2 had an almost identical structure as **1** and sinumaximol G (**6**) [16]. The difference between those two compounds was the deshielding of the H_3_-19 methyl protons (*δ*_H_ 1.34), which indicated that the configuration of the methyl group at C-8 is altered to the *β*-configuration. The ^1^H-^1^H NOESY correlation (Figure 3) between H_3_-19 (*δ*_H_ 1.34) and H_3_-18 (*δ*_H_ 1.83) further confirmed this change, and hence compound **2** was identified as a new natural product, which we named 8-*epi*- sinumaximol G (**2**). 

Compound **3** was isolated as colorless oil. HRESIMS analysis (Figure S16) resulted in *m/z* 393.2264 [M + H]^+^ ion (calcd *m/z* 393.2272), giving a molecular formula of C_22_H_32_O_6_. The 1D NMR spectral analyses (Table 2) together with the multiplicity-edited HSQC spectrum of compound **3** showed remarkable similarity to that of compound **1** (Figures S17–S20). The difference between the two compounds was the change in the location of the acetyl moiety, which HMBC correlations confirmed (Figure 2 and Figure S21) of H_3_-20/C-1’ and H_3_-2’/C-12, and NOESY correlation (Figure 3 and Figure S22) between H_3_-20 (*δ*_H_ 1.56) and H_3_-2’ (*δ*_H_ 2.0). Changes in the ^1^H NMR chemical shifts of H-7 (*δ*_H_ 3.39) and ^13^C NMR of C-12 (*δ*_C_ 82.3), along with the NOESY (Figure 3) correlations between H-3 (*δ*_H_ 4.85) and both H-7 (*δ*_H_ 3.39) and H_3_-2′ (*δ*_H_ 2.00), indicated that H-7 and the acetyl moiety were both in α-orientation. In addition, agreement of the ^1^H NMR and ^13^C NMR chemical shifts at C-2 (*δ*_H_ 5.43, *δ*_C_ 79.3) and C-8 (*δ*_C_ 74.9) with that of **6** and the previously reported sinumaximol G (**1**), together with the NOESY correlations between H_3_-19 (*δ*_H_ 1.54) and H-7 (*δ*_H_ 3.39), and between H_3_-18 (*δ*_H_ 1.82) and H-2 (*δ*_H_ 5.43), suggested an *β*-orientation of H-2 and α-orientation of H-19. Therefore, compound **3** was identified as a new natural product which we named 12-acetyl-7, 12-*epi*- sinumaximol G (**3**).

Compound **4** was obtained as a colorless solid. Its molecular formula was deduced as C_20_H_28_O_4_ based on the HRESIMS [M + H]^+^ ion at *m/z* 333.2035 (calcd *m/z* 333.2066) (Figure S23). The ^1^H and ^13^C NMR spectral data (Table 1 and Table 2), together with the 2D spectral data of compound **4** (Figures S24–S28), was closely related to that of sarcophine (**7**) [18]. HMBC correlations (Figure 2) between H-10 (*δ*_H_ 5.76) and C-9 (*δ*_C_ 42.8)/C-11(*δ*_C_ 140.4)/C-12 (δ_C_ 37.1), H-13 (*δ*_H_ 1.78, 1.80), and C-1 (*δ*_C_ 161.7)/C-11 (*δ*_C_ 140.4)/C-12 (*δ*_C_ 37.1)/C-14 (*δ*_C_ 23.8), and H-20 (*δ*_H_ 1.38) and C-11 (*δ*_C_ 140.4)/C-12 (*δ*_C_ 37.1)/C-13 (*δ*_C_ 40.7) revealed the presence of a double bond at C-10/C-11 and it also indicated the presence of an oxygenated quaternary carbon at C-12. The NOESY correlations (Figure 3 and Figure S29) between the methyl protons H_3_-18 (*δ*_H_ 1.78) and both H-2 (*δ*_H_ 5.43) and H-7 (*δ*_H_ 3.53), suggested a *β*-orientation of both H-2 and H-7. Similarly, NOESY correlations between H-7 (*δ*_H_ 3.53) and H_3_-19 (*δ*_H_ 1.31) indicated a *β*-orientation of the methyl group at C-8. Additionally, the NOESY correlations between H-3 (*δ*_H_ 4.96) and H_3_-20 (*δ*_H_ 1.38) suggested the methyl group at C-12 to be in *α*-orientation. Therefore, compound **4** was identified as a new natural product, to which we gave the name 12-hydroxysarcoph-10-ene (**4**).

Compound **5** was obtained as colorless oil. The molecular formula was deduced as C_20_H_28_O_4_ based on HRESIMS [M + H]^+^ ion at *m/z* 333.2035 (calcd *m/z* 333.2066) (Figure S30), which indicated seven degrees of unsaturation. The ^1^H and ^13^C NMR spectral data (Table 1 and Table 2; Figures S31–S33) were in harmony with the skeleton of 8-*epi*-sarcophinone that was previously isolated from the Soft Coral *Sorcophytum glaucum* [19]. Considering ^13^C NMR chemical shifts, the following functional groups were identified; (i) an α,β-unsaturated-γ-lactone functionality: *δ*_C_ 124.0 (C-15), *δ*_C_ 163.4 (C-1) *δ*_C_ 175.1 (C-16). (ii) olefinic carbons at *δ*_C_ 120.2/144.0 (C-3/C-4) and *δ*_C_ 123.9/135.1 (C-11/C-12), and (iii) a ketone functional group at *δ*_C_ 213.7 (C-7) and alcohol functionalities at *δ*_C_ 79.3 (C-2) and *δ*_C_ 78.2 (C-8). The COSY spectrum (Figure S34) revealed the coupling of four hydrocarbon regions common to sarchophine skeletons: *δ*_H_ 5.44 (d, *J* = 12.0 Hz, H-2) and 4.95 (d, *J* = 12.0 Hz, H-3); *δ*_H_ 2.22 /2.47 (m, H_2_-5) and 2.76 /2.93 (m, H_2_-6); *δ*_H_ 1.98 (m, H_2_-9), 2.20/2.44 (m, H2-10) and 4.70 (br. t, *J* = 6 Hz, H-11) and *δ*_H_ 1.81/1.95 (m, H_2_-13) and 2.21/2.44 (m, H_2_-14). HMBC correlations (Figure 2 and Figure S35) between H-2 and *δ*_C_ 163.4 (C-1), H-2 and *δ*_C_ 120.2 (C-3), H-2 and *δ*_C_ 144.0 (C-4), H-3 and *δ*_C_ 17.8 (C-18), H_3_-18 and *δ*_C_ 120.2 (C-3), H_3_-18 and *δ*_C_ 31.5 (C-5), H_2_-5 and *δ*_C_ 144.0 (C-4), H_2_-5 and *δ*_C_ 34.2 (C-6) and H_2_-6/H_2_-5 and *δ*_C_ 213.7 (C-7); these correlations confirmed the linkage from C-1 to C-7. Additional correlations between H_3_-19 and *δ*_C_ 213.7 (C-7), H_2_-9, and *δ*_C_ 78.2 (C-8), H_2_-10 and *δ*_C_ 35.8 (C-9), H-11 and *δ*_C_ 25.7 (C-10), H_3_-20 and *δ*_C_ 123.9 (C-11), H_2_-10 and *δ*_C_ 135.1 (C-12), H_3_-20 and *δ*_C_ 39.4 (C-13), H_2_-13 and *δ*_C_ 163.4 (C-1), and H_2_-14 and *δ*_C_ 163.4 (C-1) confirmed the 14-membered ring skeleton. ^1^H-^1^H NOESY correlations (Figure 3 and Figure S36) between H_3_-18 (*δ*_H_ 1.89) and H-2 (*δ*_H_ 5.44), and between H_3_-19 (*δ*_H_ 1.26) and both H-3 (*δ*_H_ 4.95) and H-20 (*δ*_H_ 1.53) suggested a *β*-orientation of H-2 and a *α*-orientation of H-19. The chemical shifts of H-3 at *δ*_H_ 4.95 and H-11 at *δ*_H_ 4.70 are very characteristic for *E* configuration at the C-11/C-12 and C-3/C-4 double bonds [6,16,20], which was further confirmed by the ^1^H-^1^H NOESY correlations of the two olefinic methyls at C-4 and C-12 with H-2 and H_2_-10, respectively. Based on these data, compound **5** was considered to be a new natural product, which was named 8-hydroxy-*epi*-sarcophinone (**5**).

Compounds **6** and **7** were previously isolated from the Soft Corals *Sinularia maxima* and *Sorcophytum glaucum,* and identified as sinumaximol G (**6**) [16] and sarcophine (**7**) [18], respectively. Their structures were confirmed based on a comparison of their HRMS and NMR data with literate.

### 2.2. Antiproliferative Activity

Our previous *in vitro* screening results showed that, among several cancer cell lines, sarchophine (**7**), along with other analogs, were only active against breast (MDA-MB-231) and prostate (PC-3) cancer cell lines [6]. We consequently chose a breast carcinoma cell line (MCF-7) to assess the *in vitro* anticancer effect of the isolated compounds (**1**–**7**). All of the tested compounds induced dose-dependent cell death with IC_50_ values (Table 3) of (22.39–27.12 µg/mL). Although all of the tested compounds have significant cytotoxic potentials against MCF-7, they have slightly reduced (approx. two-fold) anticancer effects in comparison to the standard anticancer drug doxorubicin (IC_50_: 12.78 µg/mL). In the light of these results, along with the previous reports [6,16,19,20,21] on other cembrane-type diterpenoids, we can conclude that the presence of an α, β-unsaturated-γ-lactone moiety is an essential feature for the antiproliferative properties of these class of compounds. Additionally, changing in the orientation or acetylation of hydroxyl groups at C-7, C-8, and C-12 almost have no effect on their cytotoxicity. 

## 3. Materials and Methods 

### 3.1. General Experimental Procedures

Silica gel 60 (Natland, 63–200 µm) and solvent systems consisting of n-hexane-EtOAc (9.5:0.5 to 70:30) were used for column chromatography. Pre-coated silica gel plates (Merck, Darmstadt, Germany, Kieselgel 60 F_254_, 0.25 mm) were used for TLC analyses. 1% vanillin in concentrated H_2_SO_4_ was used as the visualizing reagent. LC/MS was conducted on a Thermo MS system (LTQ XL/LTQ Orbitrap Discovery) coupled to a Thermo Instruments HPLC system with Accela PDA detector: The data were processed using Xcalibur 2.0.7. ^1^H and ^13^C NMR spectra were recorded in CDCl_3_ on a JEOL ECA-600 spectrometer (600 MHz for ^1^H and 150 MHz for ^13^C, respectively). All of the chemical shifts (δ) are given in ppm units with reference to TMS as an internal standard, and coupling constants (J) are reported in Hz.

### 3.2. Extraction and Fractionation

The soft coral *Sarcophyton* sp. was collected from the Egyptian Red Sea off the coast of Hurghada (GPS coordinates N 27°15048”, E 33°4903”) at depths of 5–7 m in March 2016 and then frozen for storage. A voucher specimen (NIOF404/2016) was reserved at the National Institute of Oceanography and Fisheries, Red Sea Branch, Invertebrates Department. The frozen soft coral (700 g) was extracted four times with isopropanol. The extract was concentrated under vacuum to give 70 g raw material and then chromatographed on silica gel while using *n*-hexane/EtOAc to afford seven fractions. Only fractions 6 and 7 exhibited *in vitro* cytotoxicity against MCF-7 cell lines. In addition, LC-HRESMS dereplication results were used to prioritize those fractions, as they showed several unknown molecular formulas. Further chromatographic separation of fractions 6 and 7 on silica gel while using a gradient of *n*-hexane/EtOAc yielded compounds **1**–**7**, which were purified on Sephadex LH-20 using 10% aqueous MeCN.

7-Acetyl-8-*epi*-sinumaximol G (**1**): colorless oil; $[\alpha {]}_{D}^{\mathrm{25}}$ +4.8 (c 0.54, CH_2_Cl_2_); ^1^H NMR and ^13^C NMR data, see Table 1 and Table 2; HR-EI-MS [M + H]^+^ m/z 393.2262 (calc. 393.2272, C_22_H_32_O_6_). 

8-*epi*-Sinumaximol G (**2**): colorless oil; $[\alpha {]}_{D}^{\mathrm{25}}$ +5.4 (c 0.50, CH_2_Cl_2_); ^1^H NMR and ^13^C NMR data, see Table 1 and Table 2; HR-FAB-MS [M + H]^+^ m/z 351.2160 (calc. 351.2166, C_20_H_30_O_5_). 

12-Acetyl-7, 12-*epi*- sinumaximol G (**3**): colorless oil; $[\alpha {]}_{D}^{\mathrm{25}}$ +1.7 (c 0.58, CH_2_Cl_2_); ^1^H NMR and ^13^C NMR data, see Table 1 and Table 2; HR-EI-MS [M + H]^+^ m/z 393.2264 (calc. 393.2272, C_22_H_32_O_6_).

12-Hydroxysarcoph-10-ene (**4**): colorless oil; $[\alpha {]}_{D}^{\mathrm{25}}$ +4.6 (c 0.46, CH_2_Cl_2_); ^1^H NMR and ^13^C NMR data, see Table 1 and Table 2; HR-EI-MS [M + H]^+^ m/z 343.2840 (calc. 343.2843, C_20_H_38_O_4_). 

8-Hydroxy-*epi*-sarcophinone (**5**): colorless oil; $[\alpha {]}_{D}^{\mathrm{25}}$ +5.1 (c 0.40, CH_2_Cl_2_); ^1^H NMR and ^13^C NMR data, see Table 1 and Table 2; HR-EI-MS [M + H]^+^ m/z 343.2849 (calc. 343.2843, C_20_H_38_O_4_). 

Sinumaximol G (**6**): $[\alpha {]}_{D}^{\mathrm{25}}$ = +4.1 (c=0.48, CH_2_Cl_2_); lit. $[\alpha {]}_{D}^{\mathrm{25}}$ +3.4 (c 0.52, CH_2_Cl_2_) [16].

Sarcophine (**7**): $[\alpha {]}_{D}^{\mathrm{25}}$ +79.6 (c 0.9, CH_2_Cl_2_); lit. $[\alpha {]}_{D}^{\mathrm{25}}$ +92 (c 1.0, CHCl_3_) [6].

### 3.3. LC-HRESIMS Analysis and Dereplication

The following conditions were used for LC-HRESIMS analysis: capillary voltage 45 V, capillary temperature 260 °C, auxiliary gas flow rate 10–20 arbitrary units, sheath gas flow rate 40-50 arbitrary units, spray voltage 4.5 kV, and mass range 100–2000 amu (maximum resolution 30000). Gradient separation was achieved while using a SunFire C_18_ RP analytical HPLC column (5µm, 4.6 × 150 mm, Waters) with a mobile phase of 0–100% MeOH over 30 min. at a flow rate of 1 mL/min. Multiple available databases (SciFinder: https://sso.cas.org/as/E9dPQ/resume/as/authorization.ping, MarinLit: http://pubs.rsc.org/marinlit/, Dictionary of Natural Products: http://dnp.chemnetbase.com/faces/chemical/ChemicalSearch.xhtml, PubChem: https://pubchem.ncbi.nlm.nih.gov/, Chemspider: http://www.chemspider.com/) were used for the dereplication and annotation of known compounds.

### 3.4. In Vitro Antiproliferative Activity

The human breast cancer cell line (MCF-7) was obtained from the American Type Culture Collection (ATCC, Manassas, USA). They were seeded in 96 well microtiter plates at a concentration of 1000–2000 cells/well, 100 µL/well. After 24 h, the cells were incubated for 72 h with the compounds to be tested. Dulbecco’s Modified Eagle Medium (DMEM) with 10% foetal calf serum, sodium pyruvate, 100 U/mL penicillin, and 100 mg/mL streptomycin at 37 °C and 5% CO_2_ was used as culture medium. The medium was discarded at the end of the incubation. The cells were fixed with 150 μL cold trichloroacetic acid with 10% final concentration for 1 h at 4 °C. The plates were washed with distilled water (automatic washer Tecan, Germany) and then stained with 50 μL 0.4% Sulforhodamine B dissolved in 1% acetic acid for 30 min at room temperature in the dark. The plates were washed with 1% acetic acid to remove unbound dye and air-dried (24 h). The dye was solubilized with 150 µL/well of 10 mM tris base (pH 7.4) for 5 min. on a shaker at 1600 rpm. The optical density (OD) of each well was spectrophotometrically measured at 490 nm with an ELISA microplate reader. The mean background absorbance was automatically subtracted and the mean values of each tested compound and doxorubicin concentration was calculated. The experiment was repeated three times for each tested compound. The percentage of cell survival was calculated by using the following formula: surviving percent = [O.D. (treated cells)/O.D. (control cells)] x100. The IC_50_ values (the concentrations of compound required to produce 50% inhibition of cell growth) were also calculated.

## 4. Conclusions

The present study provides an additional chemical characterization of the Red Sea soft coral *Sarcophyton.* LC-HRESMS-based dereplication, in combination with biological activity-guided fractionation, allowed for the accelerated characterization of further new bioactive cembrane-type diterpenoids. All of the isolated compounds demonstrated moderate *in vitro* antiproliferative activity against breast cancer cell line MCF-7. This finding adds to our existing knowledge and understanding regarding the cytotoxic activity of cembranoid diterpenes, indicating that this class of compounds can be utilized as a potential scaffold for the future design of potent anticancer agents.

## Figures and Tables

**Table 1:** ^1^H NMR spectral data of 1–5 (600 MHz, CDCl_3_).

Position	1	2	3	4	5
	*δ*_H_ (*J* in Hz)	*δ*_H_ (*J* in Hz)	*δ*_H_ (*J* in Hz)	*δ*_H_ (*J* in Hz)	*δ*_H_ (*J* in Hz)
2	5.48,d,(14.0)	5.46,d,(14.0)	5.43,d,(14.0)	5.43,d(14.0)	5.44,d(15.0)
3	4.81,d,(14.0)	4.87,d,(14.0)	4.85,d,(14.0)	4.96,d,(14.0)	4.95,d(15.0)
5	1.9,m; 2.14,m	2.22,m; 2,38,m	2.21,m; 2.28,m	2.22,m; 2.40,m	2.22,m; 2.47,m
6	1.75,m; 1.90,m	1.50,m; 1.9,m	1.55,m; 2.04,m	1.62,m; 2.22,m	2.76,m; 2.93,m
7	4.82,d,(10.0)	3.22,d,(10.0)	3.39,d,(10.0)	3.53,d,(10.0)	-
9	2.20,m;2.28,m	2.25,m;2.41,m	2.53,m; 2.68m	2.20,m;2.62,m	1.98,m
10	5.48,m	5.65,m	5.61,m	5.76,m	2.44,m;2.20.m
11	5.47,d,(16.0)	5.53,d,(16.0)	5.57,d,(16.0)	5.68, d,(16.0)	4.73,t(6.0,12.0)
13	1.68,m; 1.70,m	1.70,m; 1.81,m	1.75,m; 2.23,m	1.78,m; 1.80,m	1.95,m; 1.81,m
14	2.18,m; 2.48,m	2.10,m; 2.38,m	2.00,m; 2.28,m	2.20,m; 2.32,m	2.21,m; 2.44,m
17	1.86,s	1.87,s	1.82,s	1.84,s	1.77,s
18	1.85,s	1,83,s	1.82,s	1.78,s	1.89,s
19	1.20,s	1.34,s	1.54,s	1.31,s	1.26,s
20	1.36,s	1.36,s	1.56,s	1.38,s	1.53,s
2′	2.08,s		2.00,s		

**Table 2:** NMR spectral data of 1-5 (150 MHz, CDCl_3_).

Position	1	2	3	4	5
	*δ*_C_, Type	*δ*_C_, Type	*δ*_C_, Type	*δ*_C_, Type	*δ*_C_, Type
1	161.4, qC	161.7 qC	161.4, qC	161.7, qC	163.4, qC
2	79.2, CH	79.3, CH	79.3, CH	79.1, CH	79.3, CH
3	122.9, CH	121.7, CH	121.4, CH	122.7, CH	120.2, CH
4	142.0, qC	143.8, qC	144.4, qC	142.7, qC	144.0, qC
5	35.6, CH_2_	35.5, CH_2_	35.5, CH_2_	36.1, CH_2_	31.5, CH_2_
6	25.3, CH_2_	26.4, CH_2_	27.8, CH_2_	26.7, CH_2_	34.2, CH_2_
7	74.4, CH	71.2, CH	71.7, CH	63.2, CH	213.7, qC
8	74.3, qC	74.5, qC	74.9, qC	74.9, qC	78.2, qC
9	42.9, CH_2_	42.9, CH_2_	44.6, CH_2_	42.8, CH_2_	35.8, CH_2_
10	124.5, CH	124.8, CH	125.3, CH	122.7, CH	25.7, CH_2_
11	140.0, CH	139.0, CH	136.2, CH	140.4, CH	123.9, CH
12	74.4, qC	73.1, qC	82.3, qC	73.1, qC	135.1, qC
13	39.7, CH_2_	39.6, CH_2_	36.8, CH_2_	40.7, CH_2_	39.4, CH_2_
14	23.0, CH_2_	23.7, CH_2_	22.3, CH2	23.8, CH_2_	28.7, CH_2_
15	122.9, qC	121.7, qC	123.4, qC	122.7, qC	124.0, qC
16	175.4, qC	175.2, qC	175.2, qC	175.4, qC	175.1, qC
17	9.1, CH_3_	9.3, CH_3_	9.3, CH_3_	9.3, CH_3_	8.8, CH_3_
18	16.4, CH_3_	16.3, CH_3_	16.1, CH_3_	15.5, CH_3_	17.8, CH_3_
19	24.7, CH_3_	23.7, CH_3_	25.7, CH_3_	23.3, CH_3_	28.7, CH_3_
20	25.7, CH_3_	27.8, CH_3_	23.6, CH_3_	28.9, CH_3_	15.4, CH_3_
1′	170.3, qC		189.9, qC		
2′	21.1, CH_3_		22.4, CH_3_		

**Table 3:** *In-vitro* antiproliferative activity of isolated metabolites (1–7) against MCF-7 cells.

Tested Compound	IC_50_ ± S.D. (µg/mL) ^a^
**1**	23.84 ± 0.2
**2**	26.22 ± 0.1
**3**	26.81 ± 0.2
**4**	25.28 ± 0.3
**5**	27.2 ± 0.5
**6**	24.97 ± 0.3
**7**	22.39 ± 0.2
**Doxorubicin**	12.78 ± 0.3

^a^ Values are a mean of 3 independent experiments.

## Supplementary Materials

The following are available online at https://www.mdpi.com/1660-3397/17/7/411/s1. Figures S1–S36: HRESIMS, 1D and 2D NMR spectra of isolated compounds.
