# Supplementary material for: New Antiproliferative Cembrane Diterpenes from the Red Sea Sarcophyton Species
Source: Mar Drugs. 2019 Jul 11;17(7):411. doi: 10.3390/md17070411 (PMC6669714; doi:10.3390/md17070411)
Supplement: Supplementary file 1 [file marinedrugs-17-00411-s001.pdf]

Supporting Information

New Antiproliferative Cembrane Diterpenes from the Red Sea *Sarcophyton* Species

Hossam M. Hassan [1], Mostafa E. Rateb [1,2], Marwa H. Hassan [1], Ahmed M. Sayed [3], Samah Shabana [4], Mai Raslan [5], Elham Amin [1,3], Fathy A. Behery [6,7], Osama M. Ahmed [8], Abdullatif Bin Muhsinah [9], Tobias A. M. Gulder [10,*] and Usama Ramadan Abdelmohsen [11,*]

Contents

Dereplicated metabolites from *Sarcophyton* sp.	2
The HRESIMS spectrum of **1**	3
The ^{1}H NMR (600 MHz, $CDCl_3$) spectrum of **1**	4
The ^{13}C NMR (150 MHz, $CDCl_3$) spectrum of **1**	5
The HSQC (600 MHz, $CDCl_3$) spectrum of **1**	6
The ^{1}H-^{1}H COSY (600 MHz, $CDCl_3$) spectrum of **1**	7
The HMBC (600 MHz, $CDCl_3$) spectrum of **1**	8
The expanded HMBC (600 MHz, $CDCl_3$) spectrum of **1**	9
The NOESY (600 MHz, $CDCl_3$) spectrum of **1**	10
The expanded NOESY (600 MHz, $CDCl_3$) spectrum of **1**	11
The HRESIMS spectrum of **2**	12
The ^{1}H NMR (600 MHz, $CDCl_3$) spectrum of **2**	13
The ^{13}C NMR (150 MHz, $CDCl_3$) spectrum of **2**	14
The HSQC (600 MHz, $CDCl_3$) spectrum of **2**	15
The ^{1}H-^{1}H COSY (600 MHz, $CDCl_3$) spectrum of **2**	16
The HMBC (600 MHz, $CDCl_3$) spectrum of **2**	17
The HRESIMS spectrum of **3**	18
The ^{1}H NMR (600 MHz, $CDCl_3$) spectrum of **3**	19
The ^{13}C NMR (150 MHz, $CDCl_3$) spectrum of **3**	20
The HSQC (600 MHz, $CDCl_3$) spectrum of **3**	21
The ^{1}H-^{1}H COSY (600 MHz, $CDCl_3$) spectrum of **3**	22
The HMBC (600 MHz, $CDCl_3$) spectrum of **3**	23
The NOESY (600 MHz, $CDCl_3$) spectrum of **3**	24
The HRESIMS spectrum of **4**	25
The ^{1}H NMR (600 MHz, $CDCl_3$) spectrum of **4**	26
The ^{13}C NMR (150 MHz, $CDCl_3$) spectrum of **4**	27
The HSQC (600 MHz, $CDCl_3$) spectrum of **4**	28
The ^{1}H-^{1}H COSY (600 MHz, $CDCl_3$) spectrum of **4**	29
The HMBC (600 MHz, $CDCl_3$) spectrum of **4**	30
The NOESY (600 MHz, $CDCl_3$) spectrum of **4**	31
The HRESIMS spectrum of **5**	32
The ^{1}H NMR (600 MHz, $CDCl_3$) spectrum of **5**	33
The ^{13}C NMR (150 MHz, $CDCl_3$) spectrum of **5**	34
The HSQC (600 MHz, $CDCl_3$) spectrum of **5**	35
The ^{1}H-^{1}H COSY (600 MHz, $CDCl_3$) spectrum of **5**	36
The HMBC (600 MHz, $CDCl_3$) spectrum of **5**	37
The NOESY (600 MHz, $CDCl_3$) spectrum of **5**	38

Table S1. Dereplicated metabolites from *Sarcophyton* sp.

No.	Molecular formula	Identification
1	$C_{14}H_{22}O$	Lobocalone
2	$C_{18}H_{37}NO$	NO HITS from *Sarcophyton*
3	$C_{20}H_{26}O_3$	lobophytolide C
4	$C_{20}H_{26}O_3$	Glaucopine C
5	$C_{20}H_{28}O$	Sinulobatin B
6	$C_{20}H_{28}O_2$	Kaurenolide; ent-16-Kauren-19,6*β*-olide
7	$C_{20}H_{28}O_3$	Sarcophine
8	$C_{20}H_{28}O_4$	9*β*-hydroxysarcophine
9	$C_{20}H_{30}O$	Sarcophytonin-A
10	$C_{20}H_{30}O_2$	16-Deoxysarcophine
11	$C_{20}H_{30}O_6$	Sinulariolone
12	$C_{20}H_{31}O_5N$	5α,12-Dihydroxy-1-tremulen-11-yl 2(S)-pyroglutamat
13	$C_{20}H_{31}O_4N$	lepadin C
14	$C_{20}H_{33}O_4N$	NO HITS from *Sarcophyton*
15	$C_{20}H_{33}O_6N$	NO HITS from *Sarcophyton*
16	$C_{20}H_{34}O_8$	Botcinolide
17	$C_{21}H_{36}O_4$	Sclerophytin F methyl ether
18	$C_{22}H_{30}O_5$	Lobophytol acetate
19	$C_{22}H_{42}O_4$	NO HITS from *Sarcophyton*
20	$C_{24}H_{24}O$	NO HITS from *Sarcophyton*
21	$C_{24}H_{24}O_7$	NO HITS from *Sarcophyton*
22	$C_{24}H_{36}O_4$	Secosarcophinolide
23	$C_{24}H_{46}O_7$	NO HITS from *Sarcophyton*
24	$C_{26}H_{24}O$	NO HITS from *Sarcophyton*
25	$C_{26}H_{24}O_2$	NO HITS from *Sarcophyton*
26	$C_{26}H_{28}O_3$	NO HITS from *Sarcophyton*
27	$C_{26}H_{42}O_4$	Dihydroxy-24-methylscalaran-25,24-olide
28	$C_{26}H_{44}O_7$	24-methyl-bisnor-cholest-22-ene-heptol
29	$C_{26}H_{47}O_5N_3$	NO HITS from *Sarcophyton*
30	$C_{26}H_{48}O_2$	NO HITS from *Sarcophyton*
31	$C_{28}H_{44}O$	NO HITS from *Sarcophyton*
32	$C_{28}H_{46}O_2$	NO HITS from *Sarcophyton*
33	$C_{28}H_{46}O_4$	3β,5α,6β,9α-Tetrahydroxy-ergosta-7,22-diene
34	$C_{28}H_{51}O_5N_3$	NO HITS from *Sarcophyton*
35	$C_{32}H_{44}O_8$	NO HITS from *Sarcophyton*
36	$C_{47}H_{60}O$	NO HITS from *Sarcophyton*

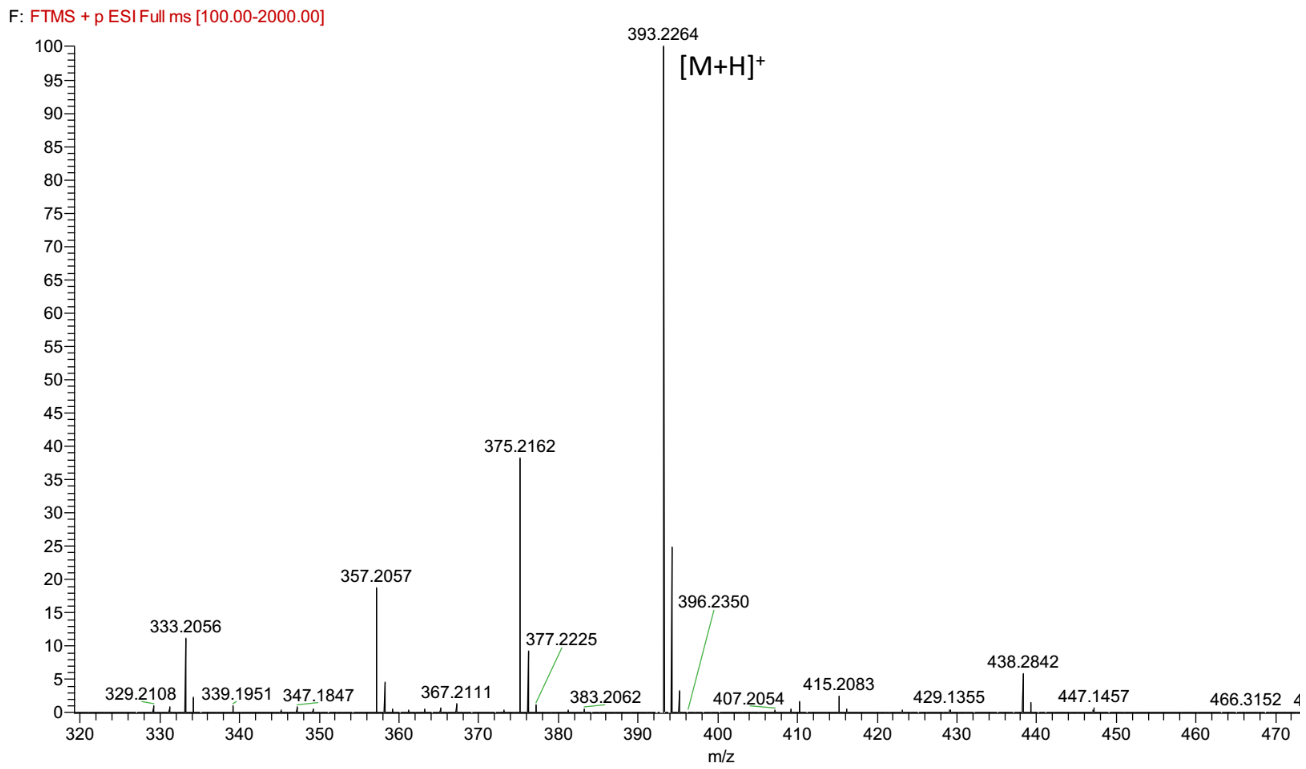

Fig.S1. The HRESIMS spectrum of **1**.

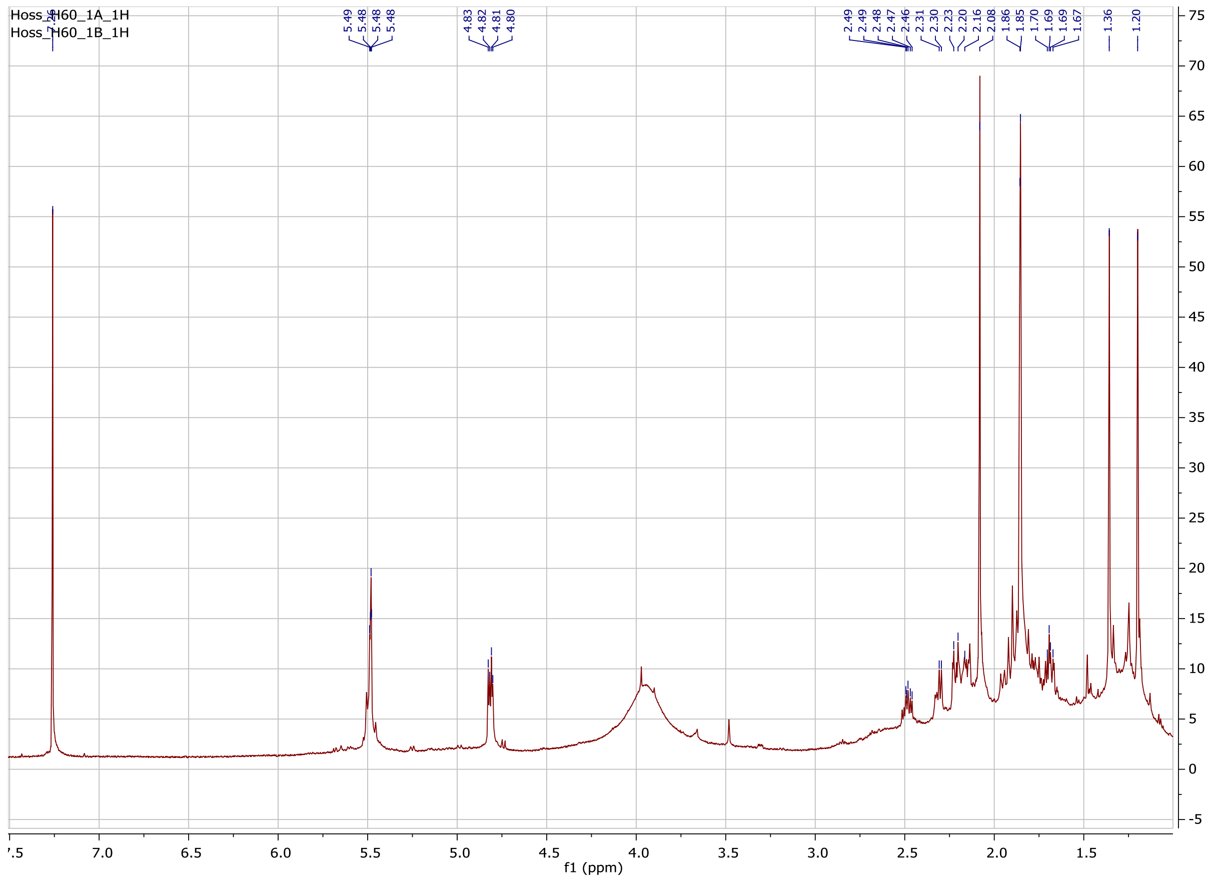

Fig.S2. The ^{1}H NMR (600 MHz, $CDCl_3$) spectrum of **1.**

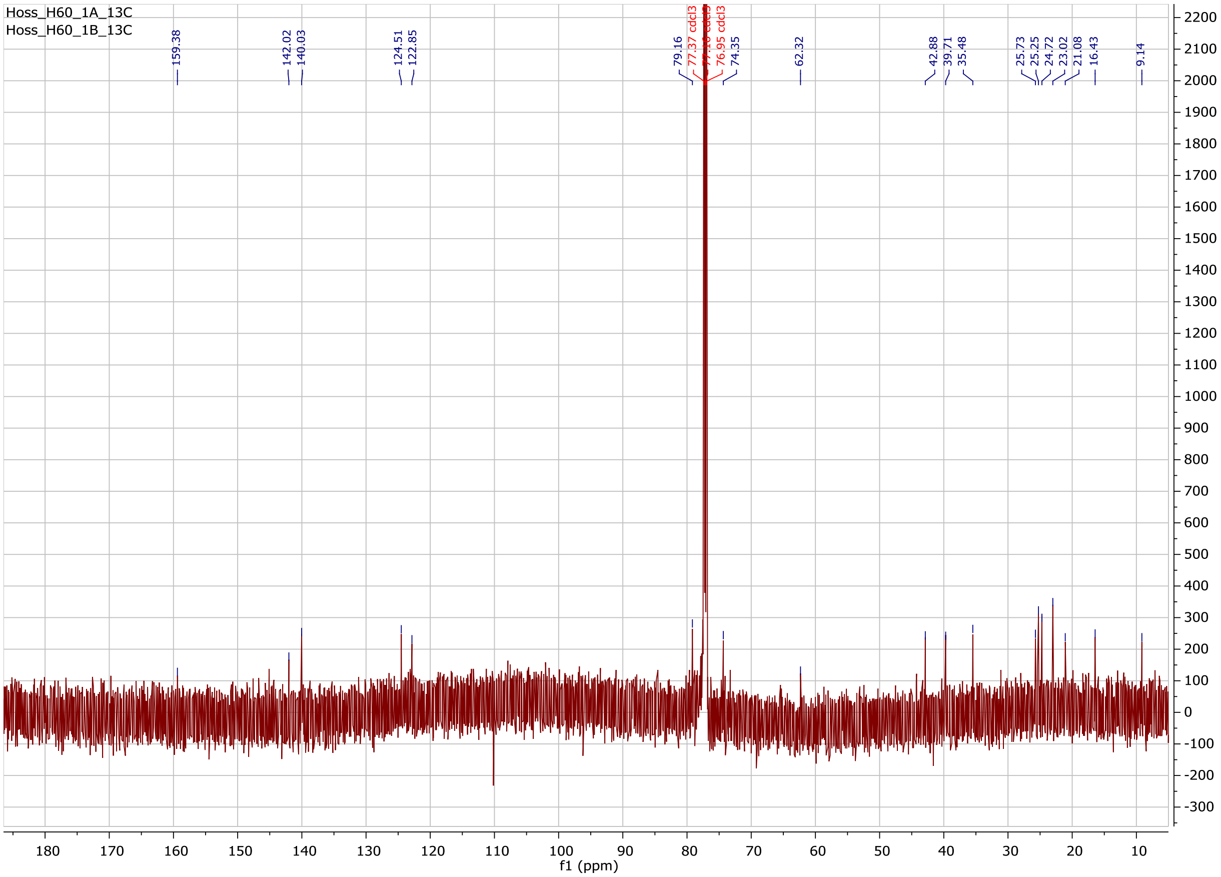

Fig.S3.The ^{13}C NMR (150 MHz, $CDCl_3$) spectrum of **1**

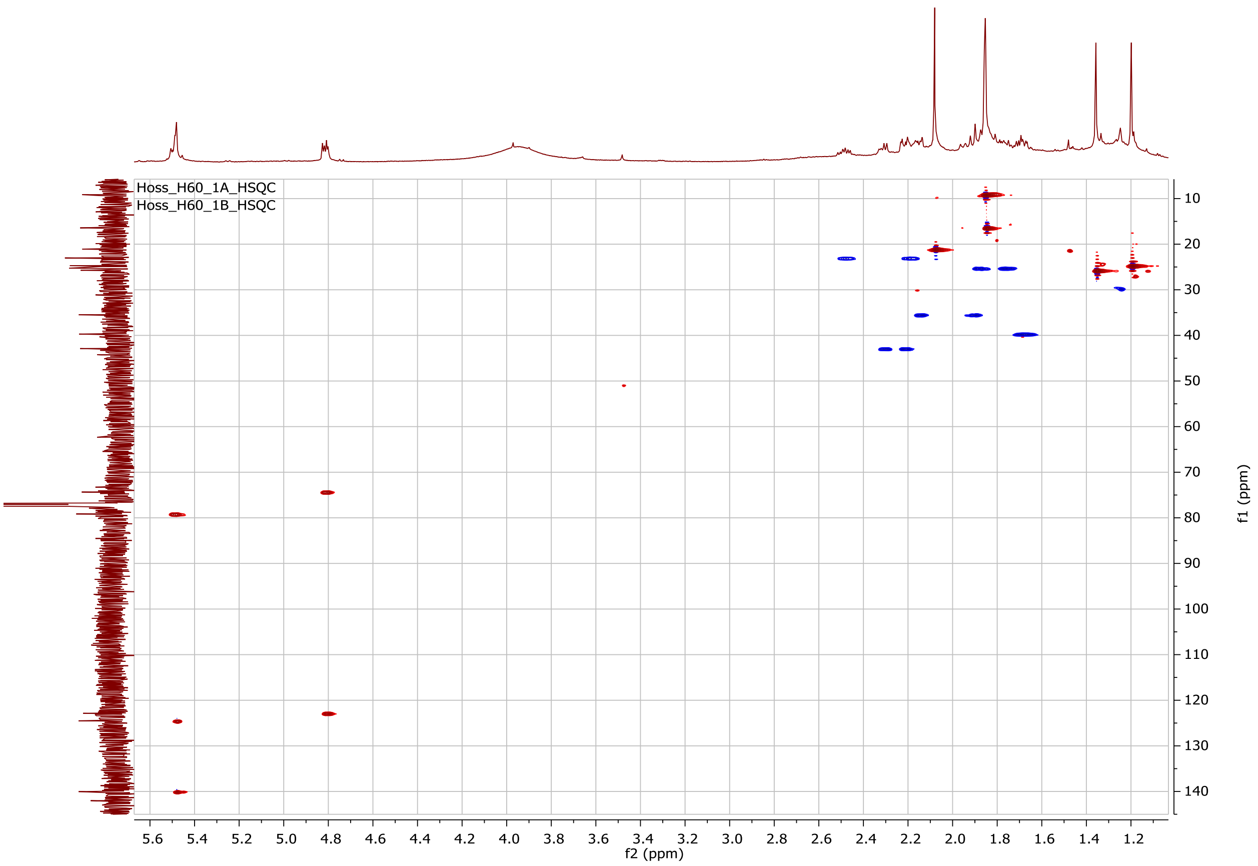

Fig.S4.The HSQC (600 MHz, $CDCl_3$) spectrum of **1**

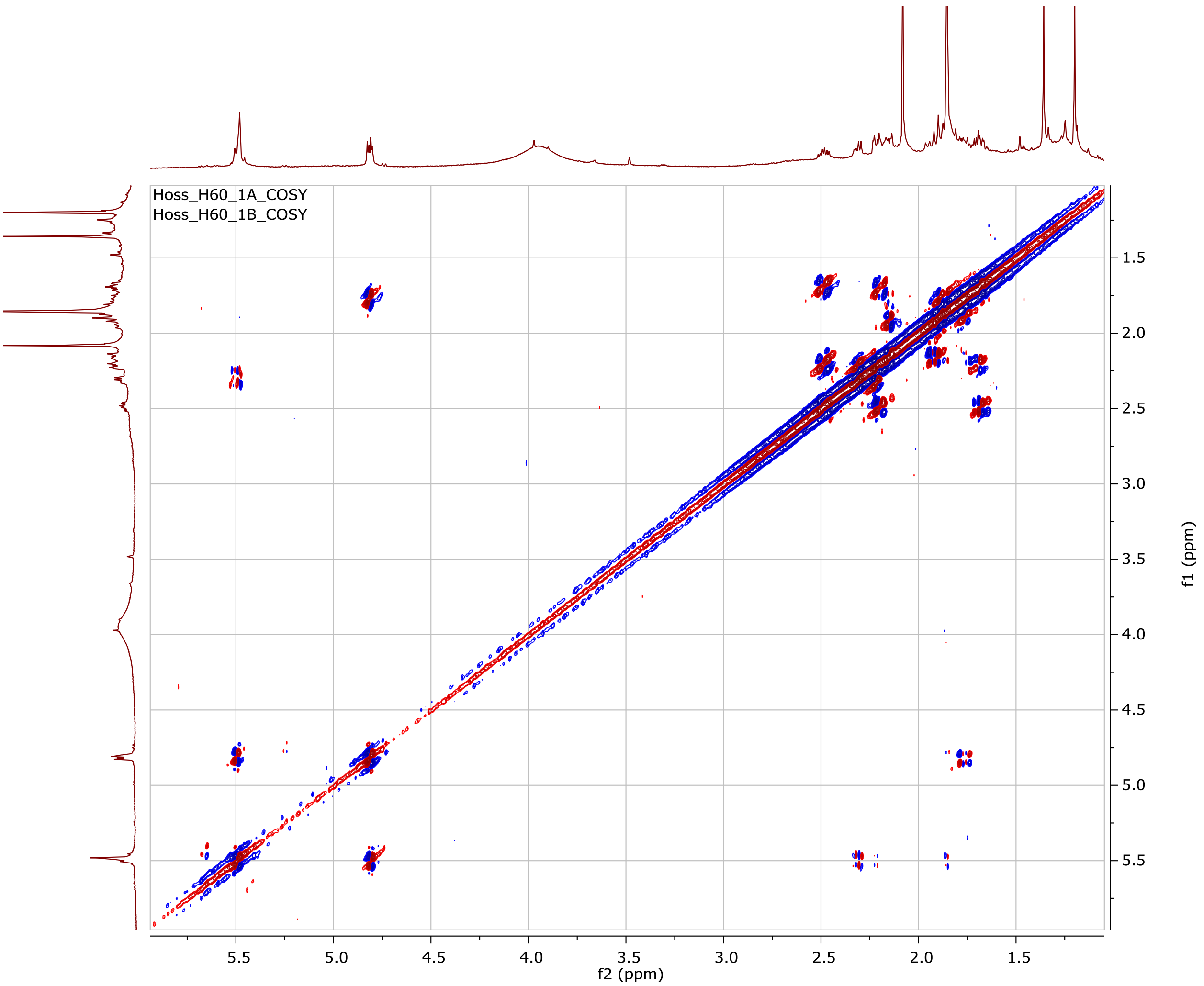

Fig.S5.The ^{1}H-^{1}H COSY (600 MHz, $CDCl_3$) spectrum of **1**

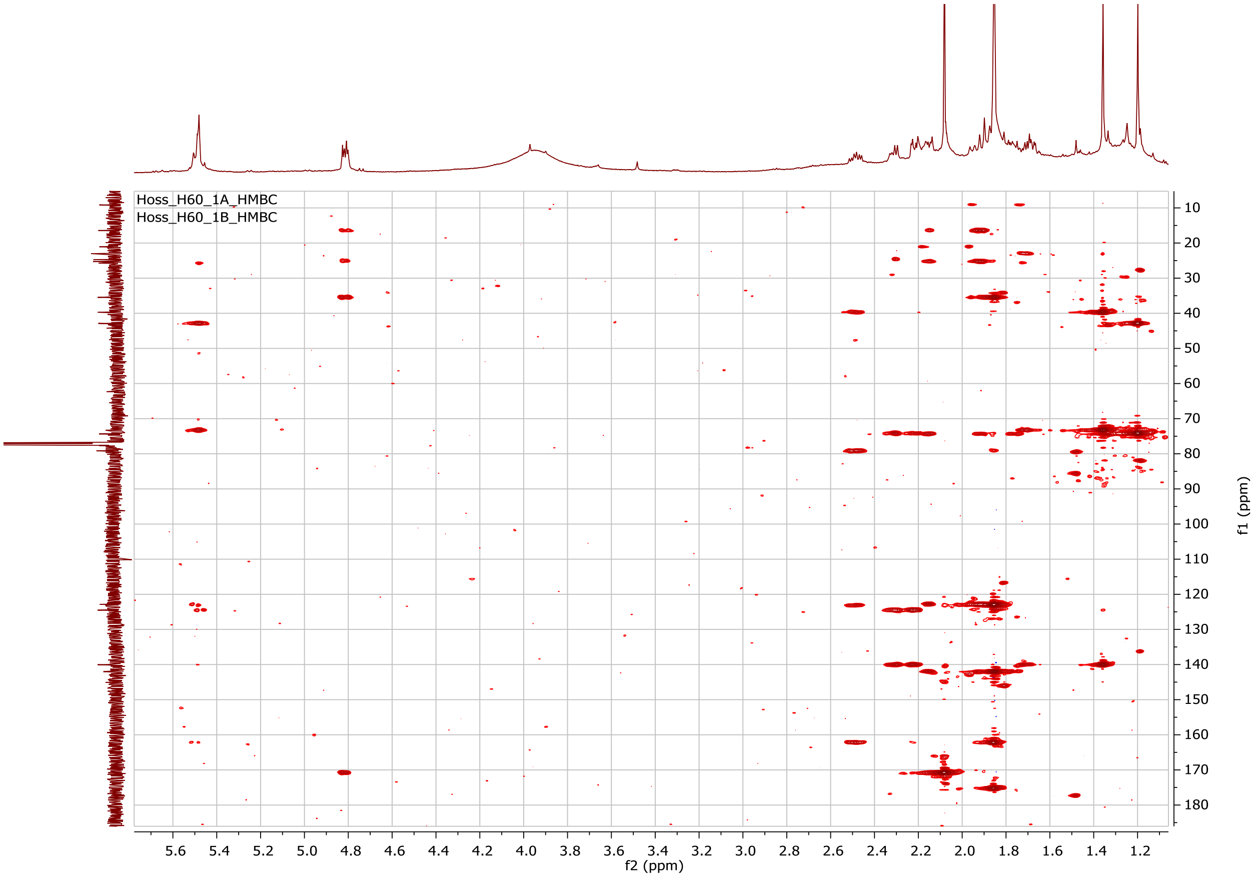

Fig.S6.The HMBC (600 MHz, $CDCl_3$) spectrum of **1**

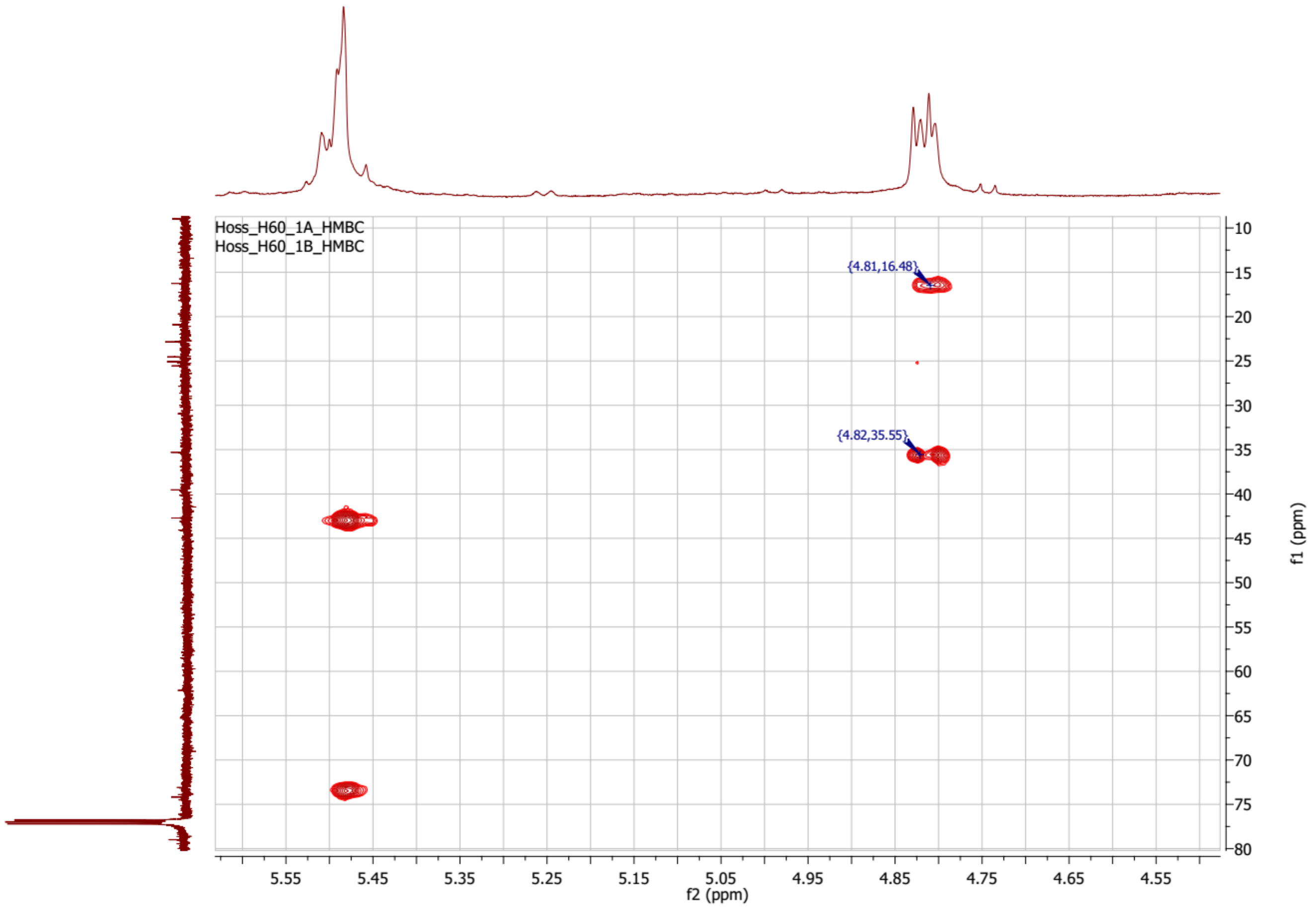

Fig.S7.The expanded HMBC (600 MHz, $CDCl_3$) spectrum of **1**

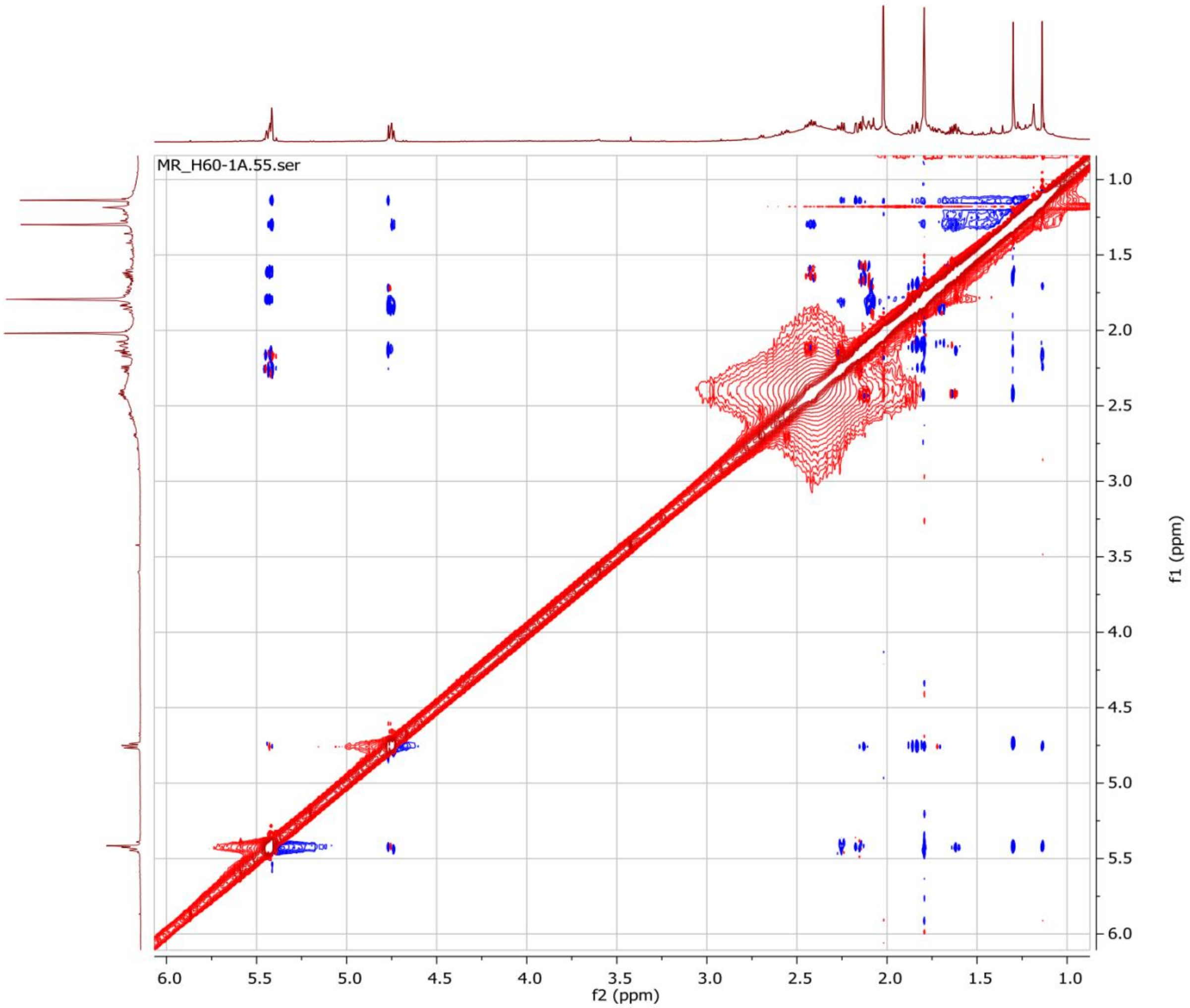

Fig.S8. The NOESY (600 MHz, $CDCl_3$) spectrum of **1**

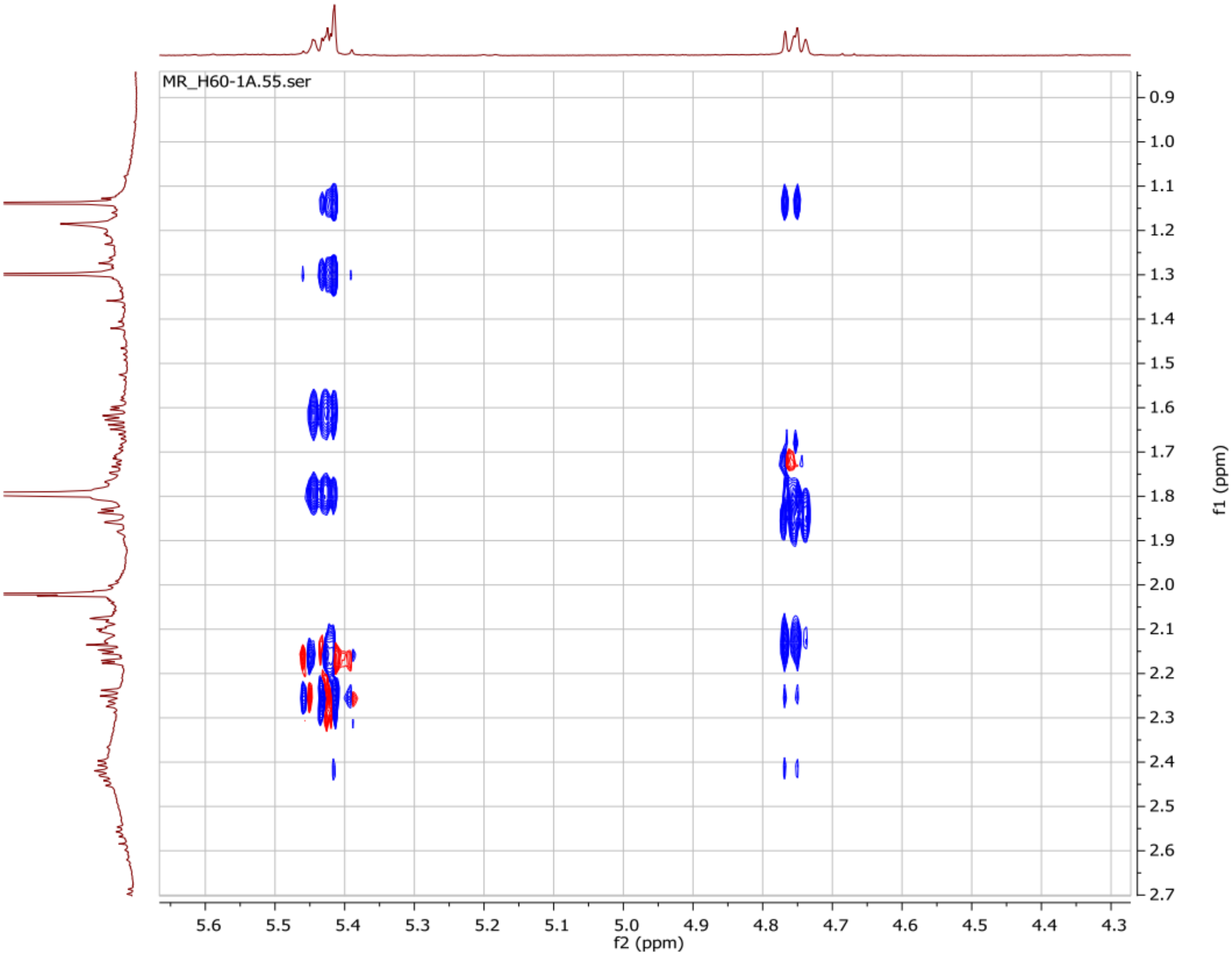

Fig.S9. The expanded NOESY (600 MHz, $CDCl_3$) spectrum of **1**

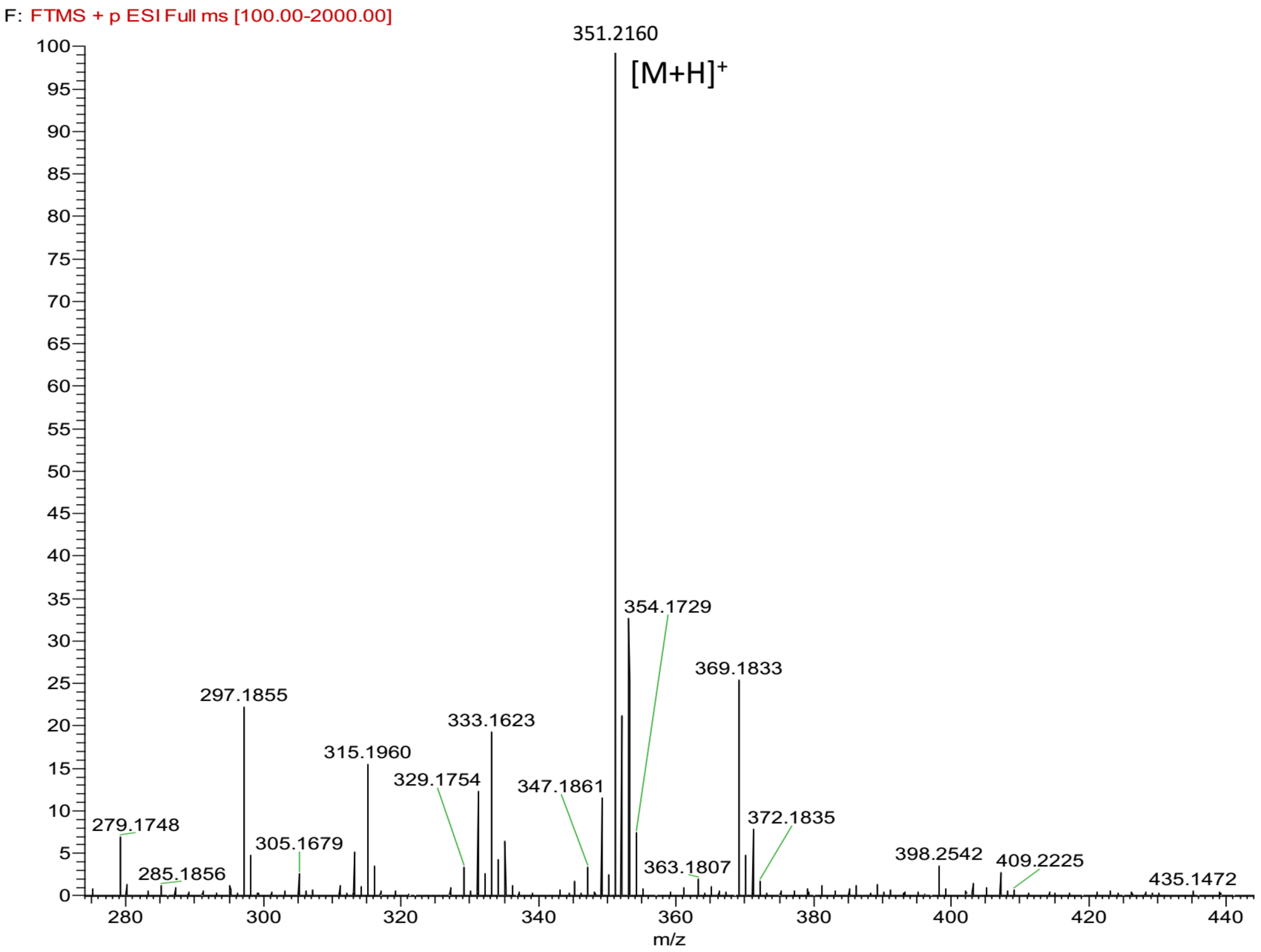

Fig.S10. The HRESIMS spectrum of **2**.

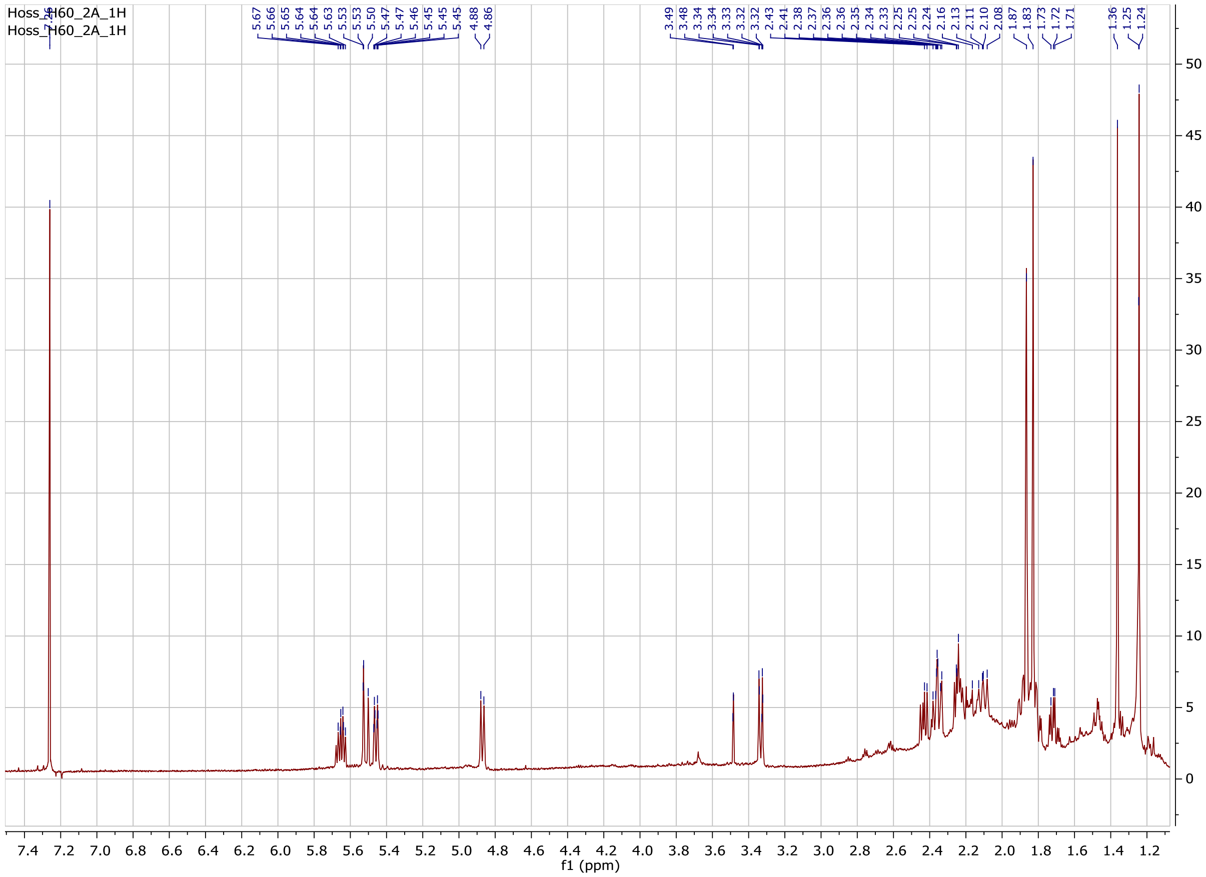

Fig.S11.The ^{1}H NMR (600 MHz, $CDCl_3$) spectrum of **2**

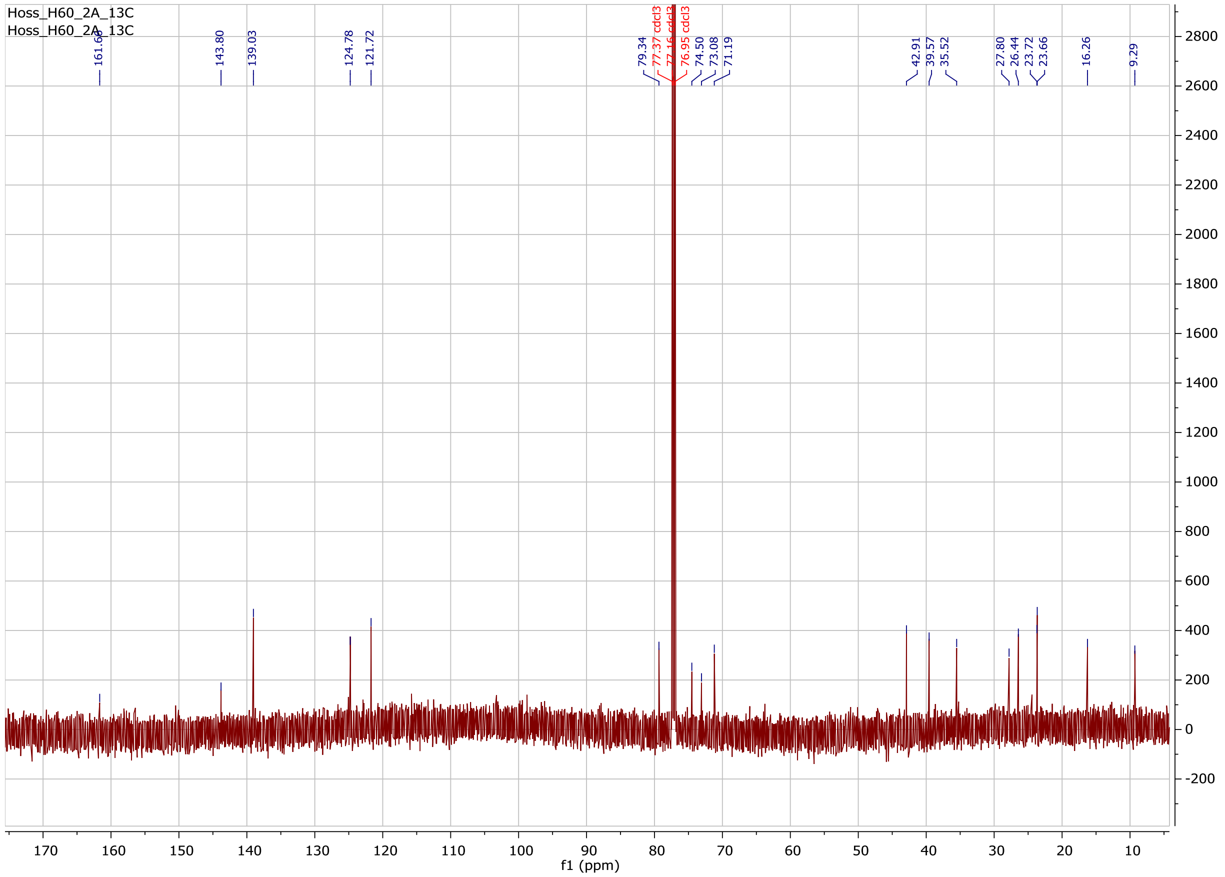

Fig.S12.The ^{13}C NMR (150 MHz, $CDCl_3$) spectrum of **2**

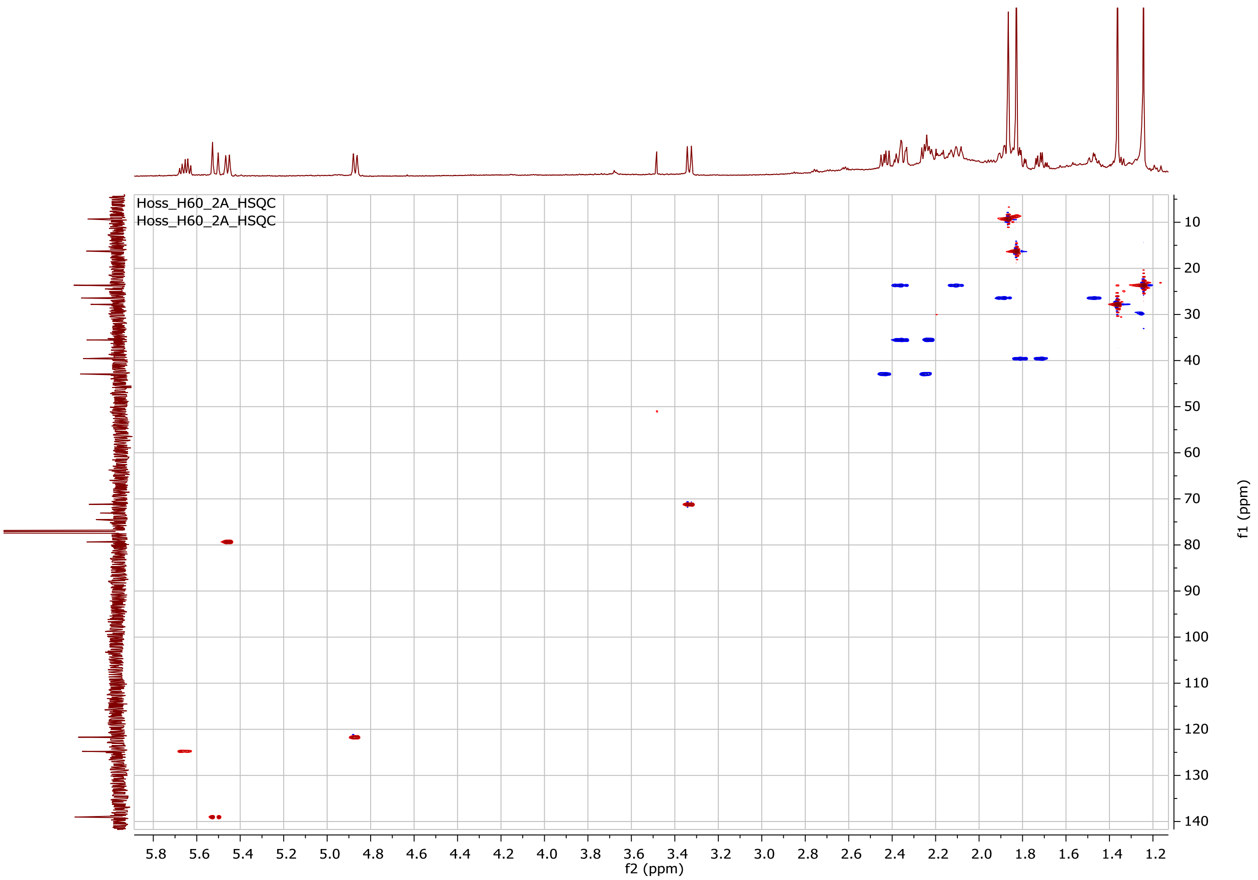

Fig.S13.The HSQC (600 MHz, $CDCl_3$) spectrum of **2**

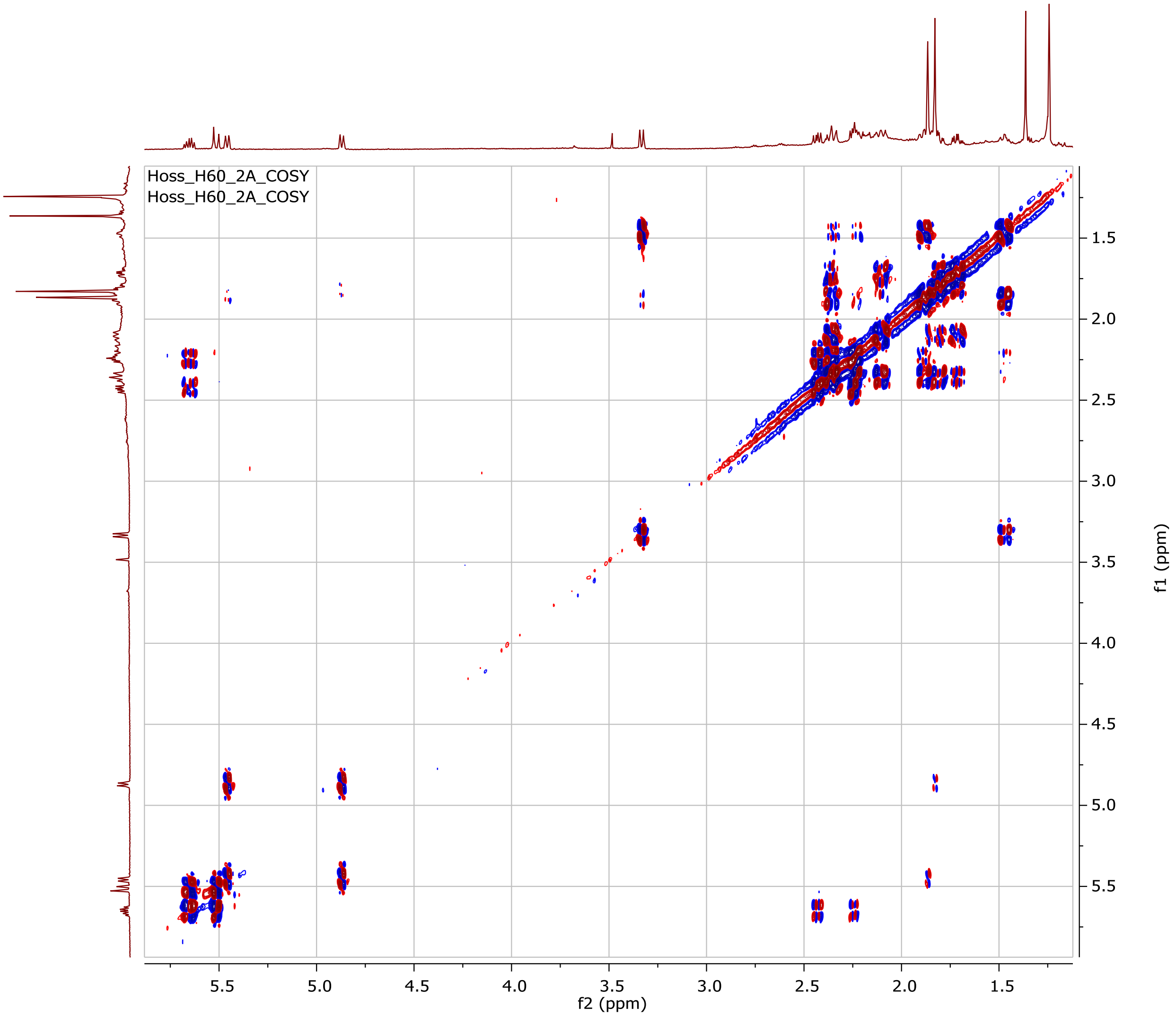

Fig.S14.The ^{1}H-^{1}H COSY (600 MHz, $CDCl_3$) spectrum of **2**

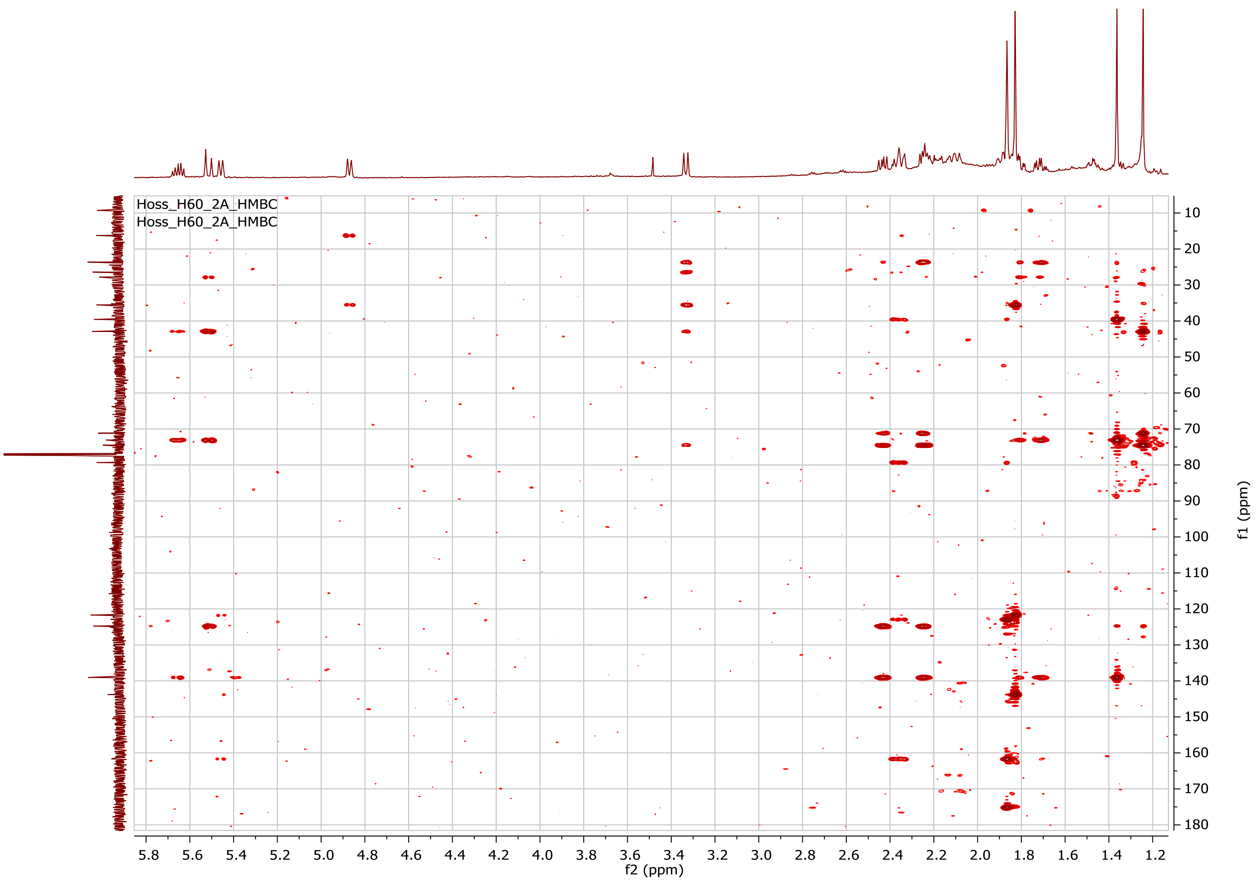

Fig.S15.The HMBC (600 MHz, $CDCl_3$) spectrum of **2**

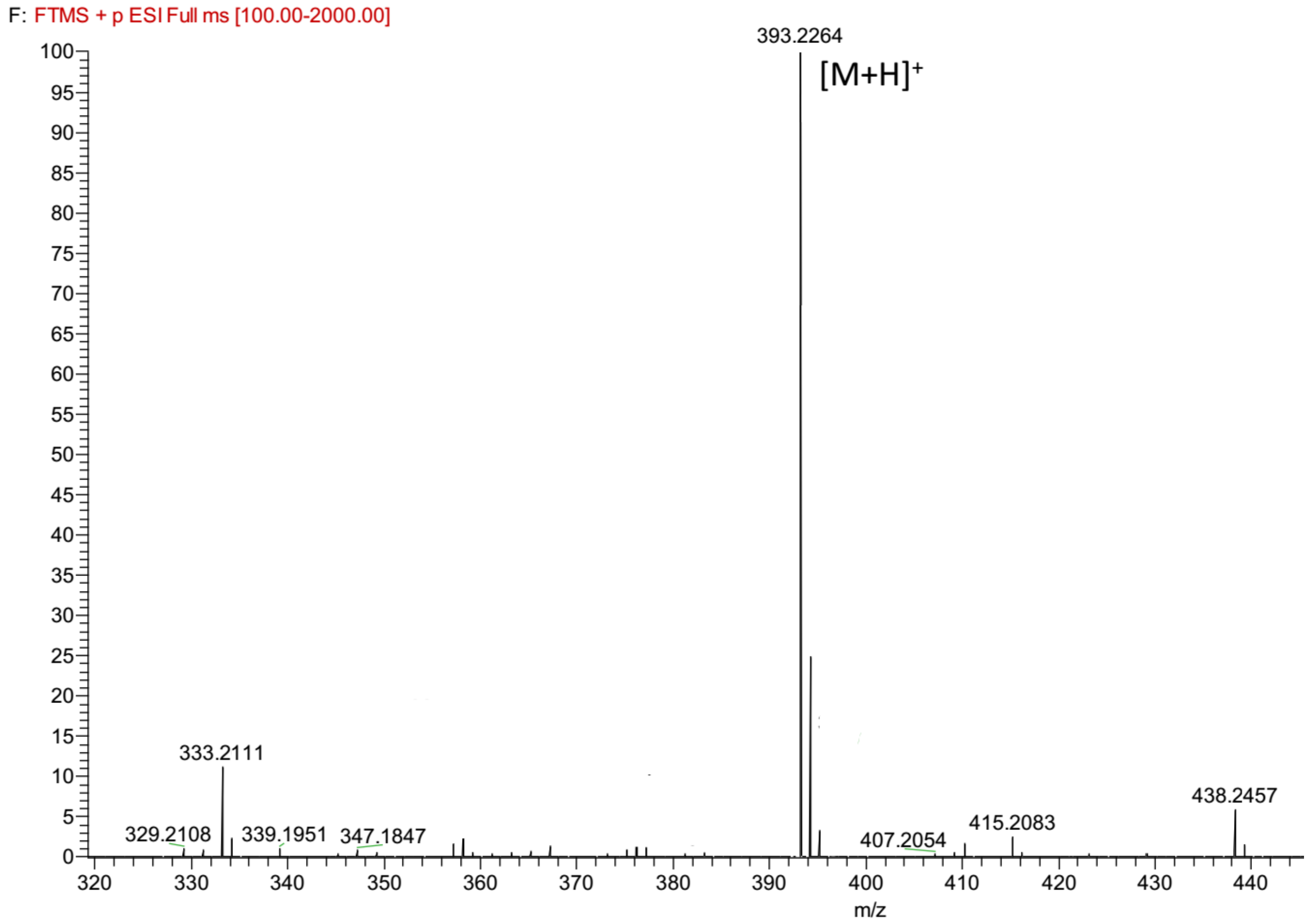

Fig.S16. The HRESIMS spectrum of **3**.

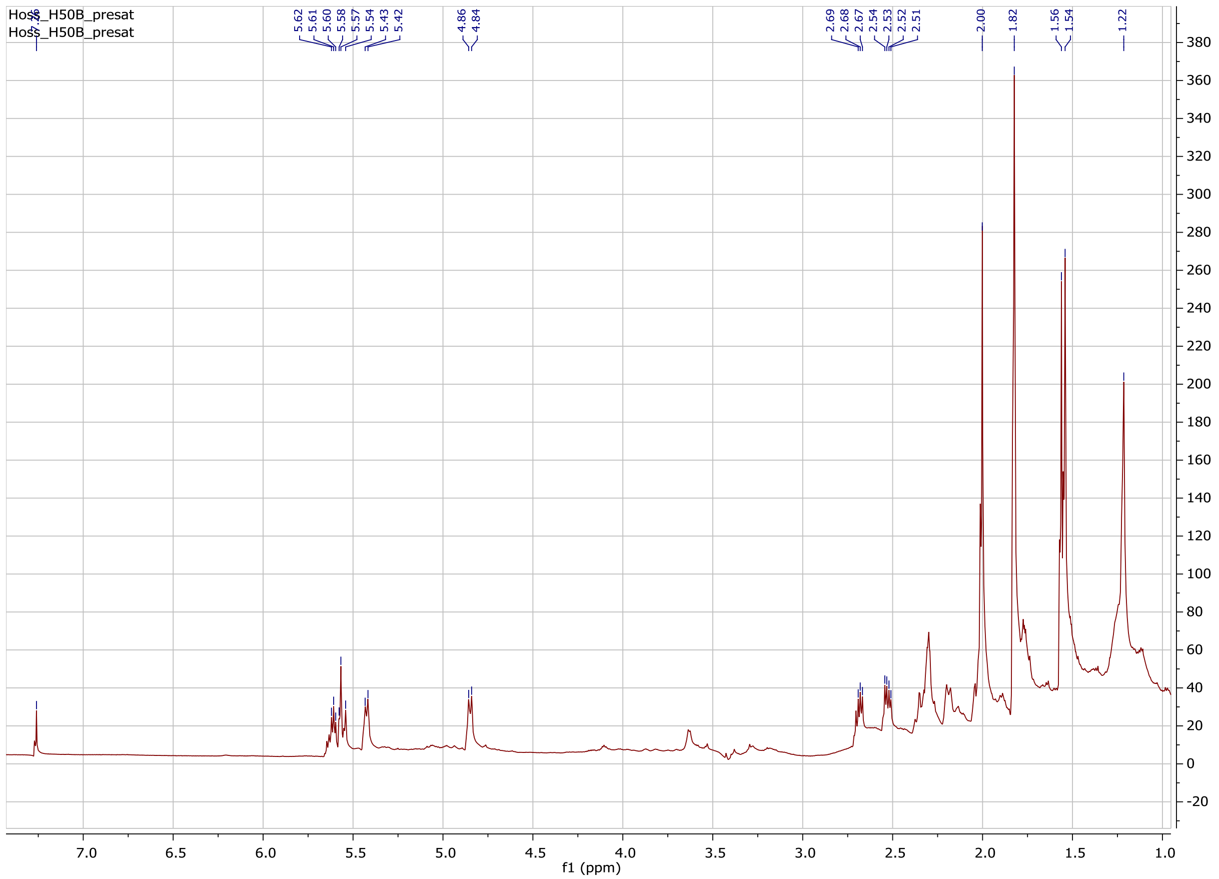

Fig.S17.The ^{1}H NMR (600 MHz, $CDCl_3$) spectrum of **3**

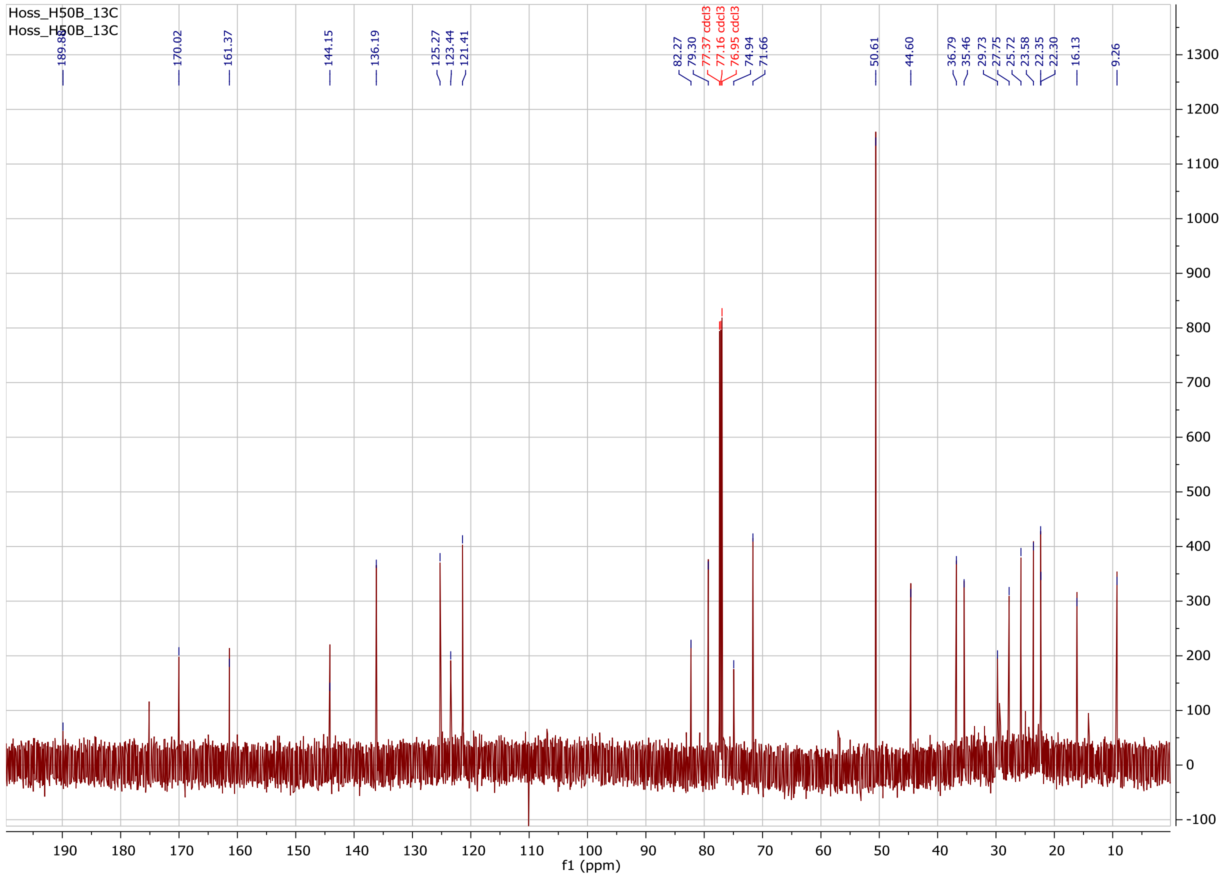

Fig.S18.The ^{13}C NMR (150 MHz, $CDCl_3$) spectrum of **3**

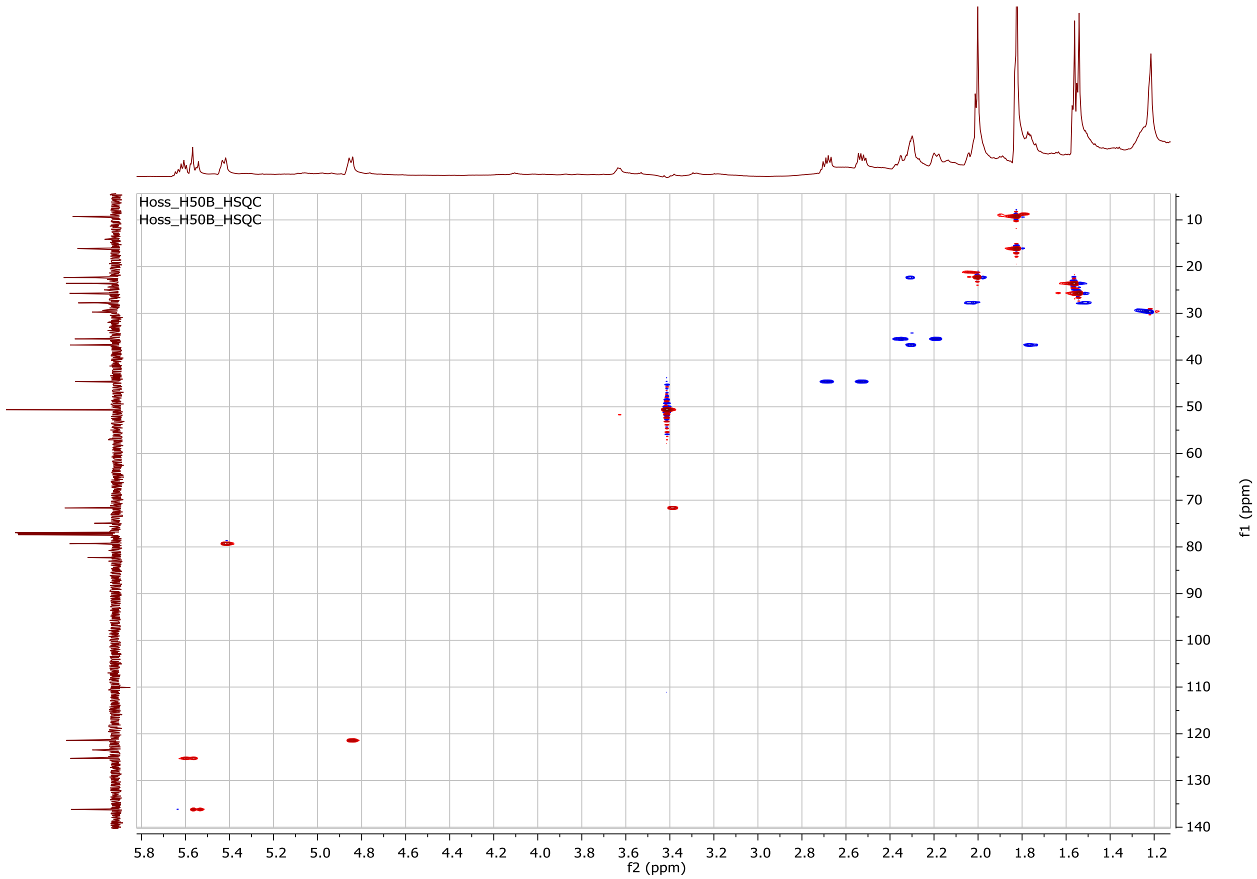

Fig.S19.The HSQC (600 MHz, $CDCl_3$) spectrum of **3**

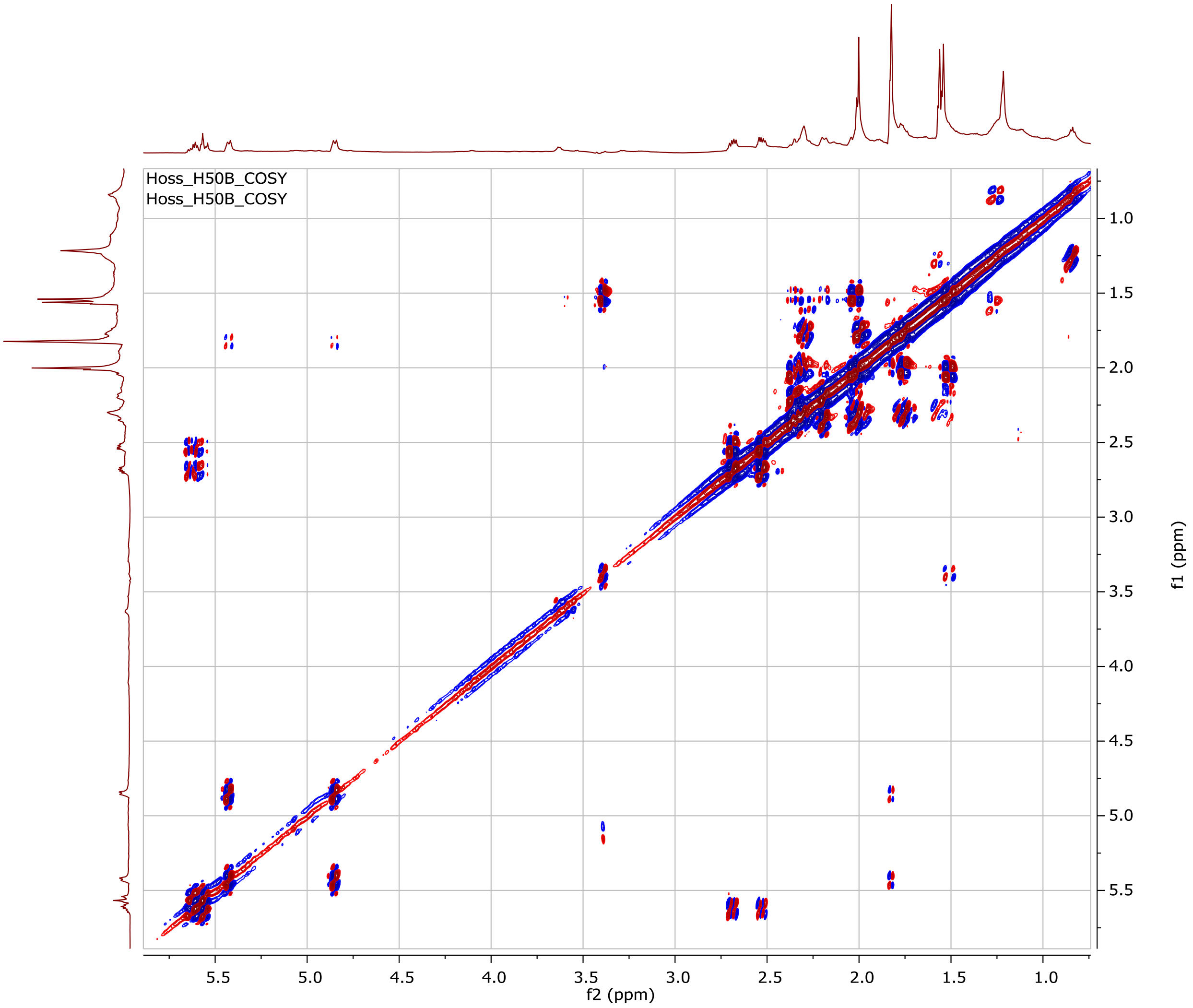

Fig.S20.The ^{1}H-^{1}H COSY (600 MHz, $CDCl_3$) spectrum of **3**

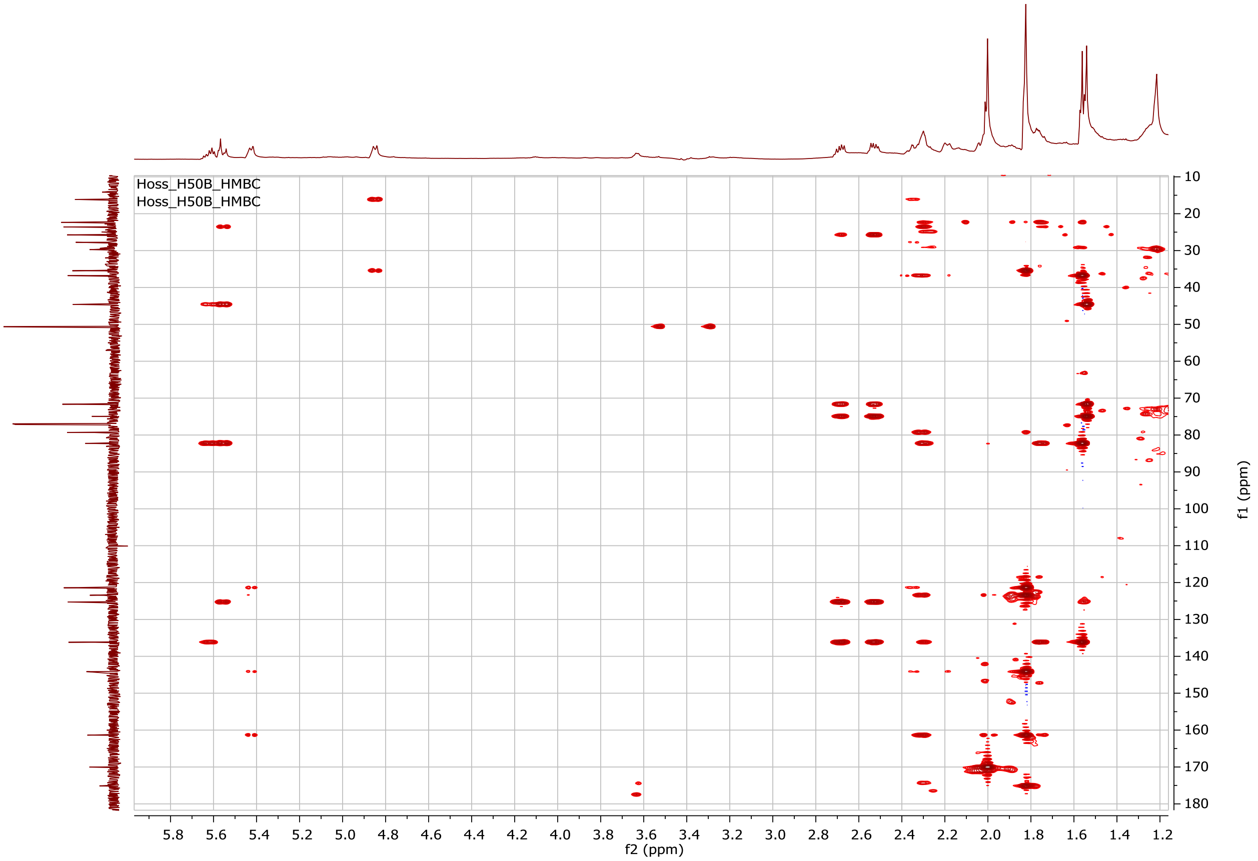

Fig.S21.The HMBC (600 MHz, $CDCl_3$) spectrum of **3**

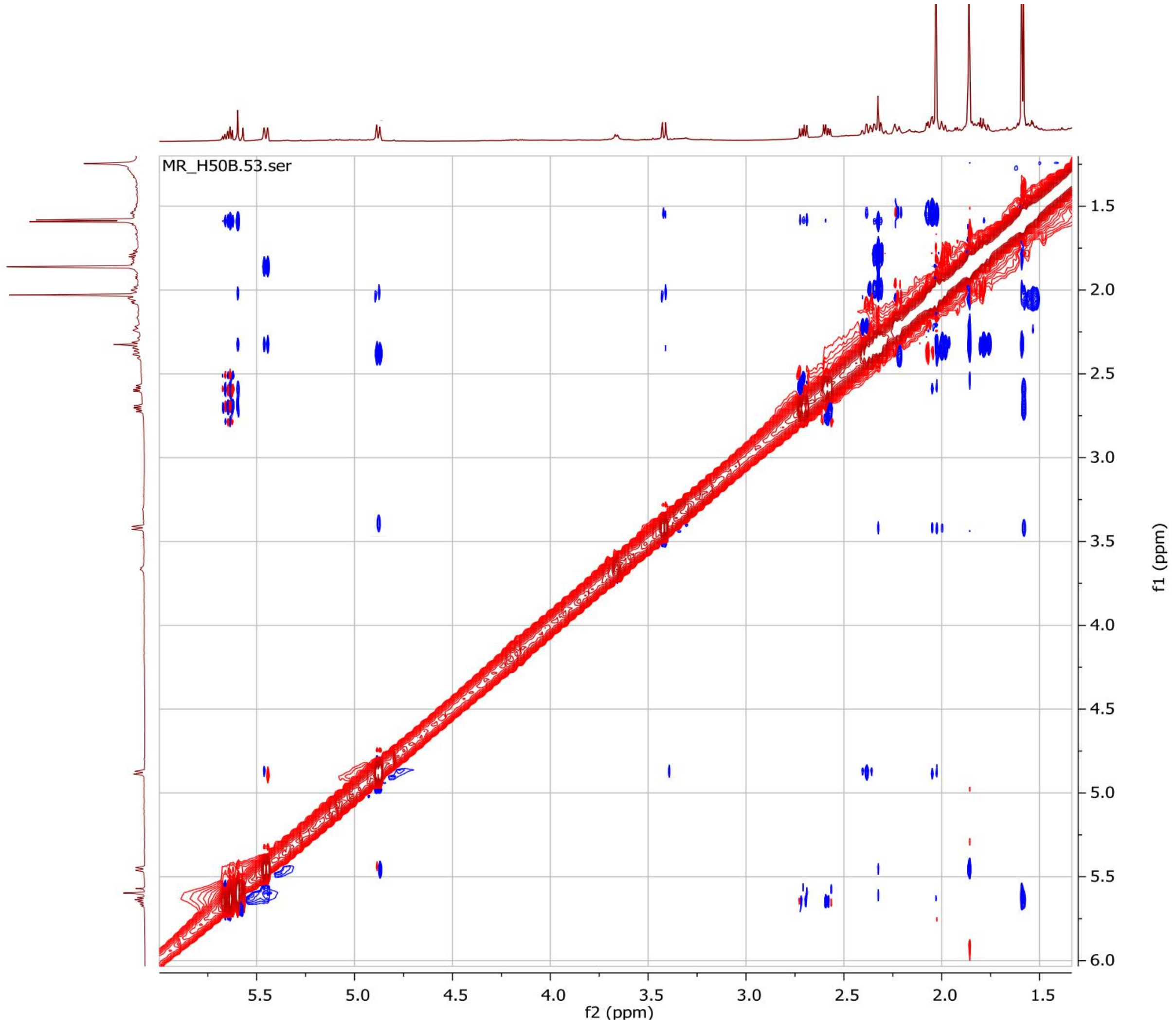

Fig.S22.The NOESY (600 MHz, $CDCl_3$) spectrum of **3**

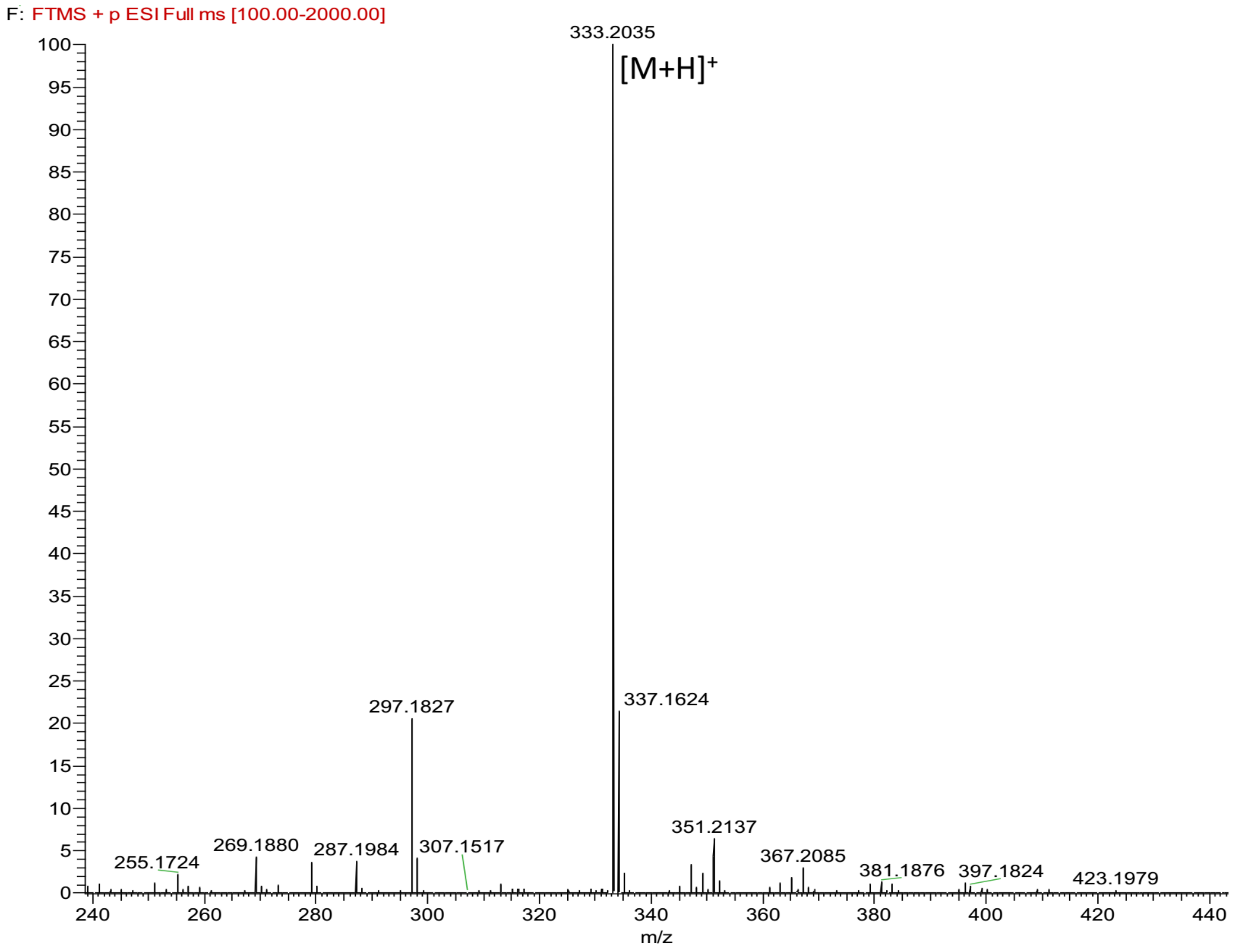

Fig.S23. The HRESIMS spectrum of **4**.

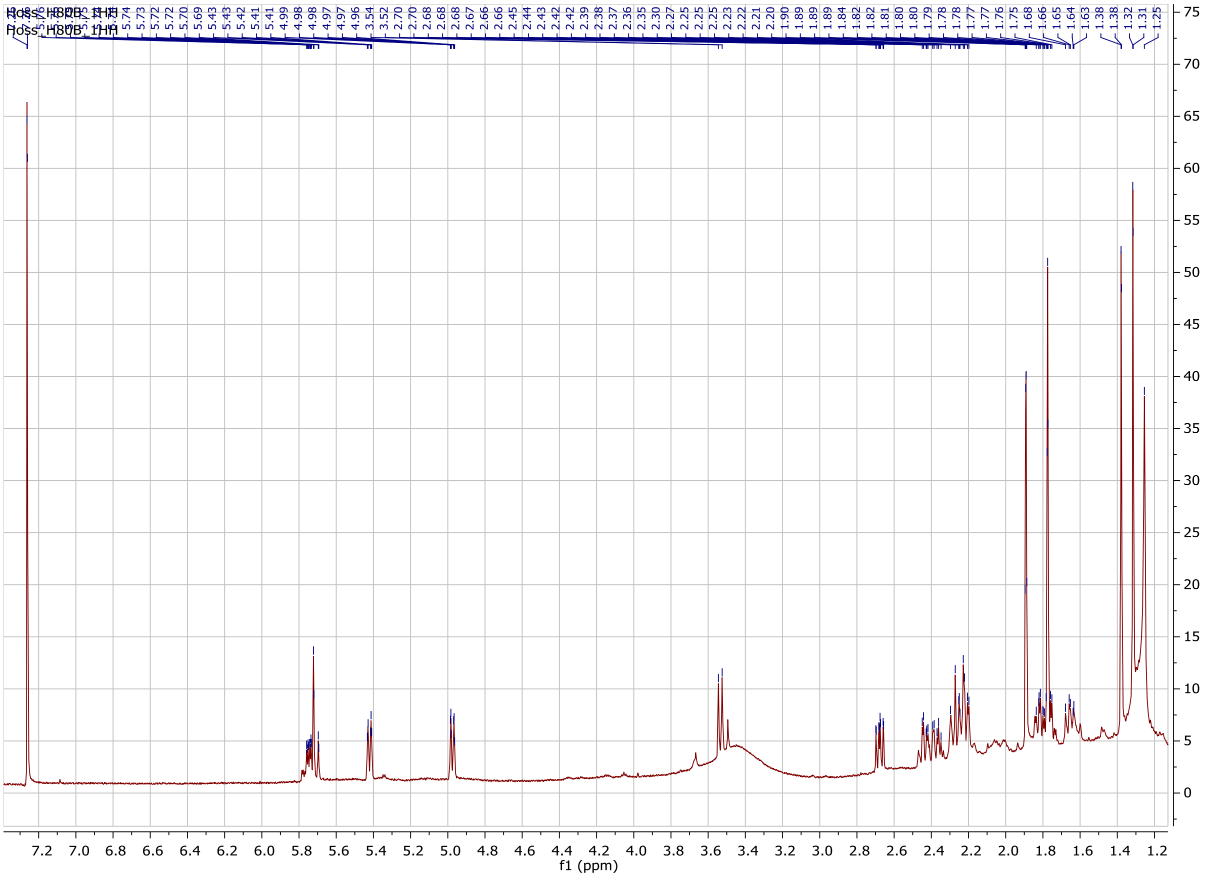

Fig.S24.The ^{1}H NMR (600 MHz, $CDCl_3$) spectrum of **4**

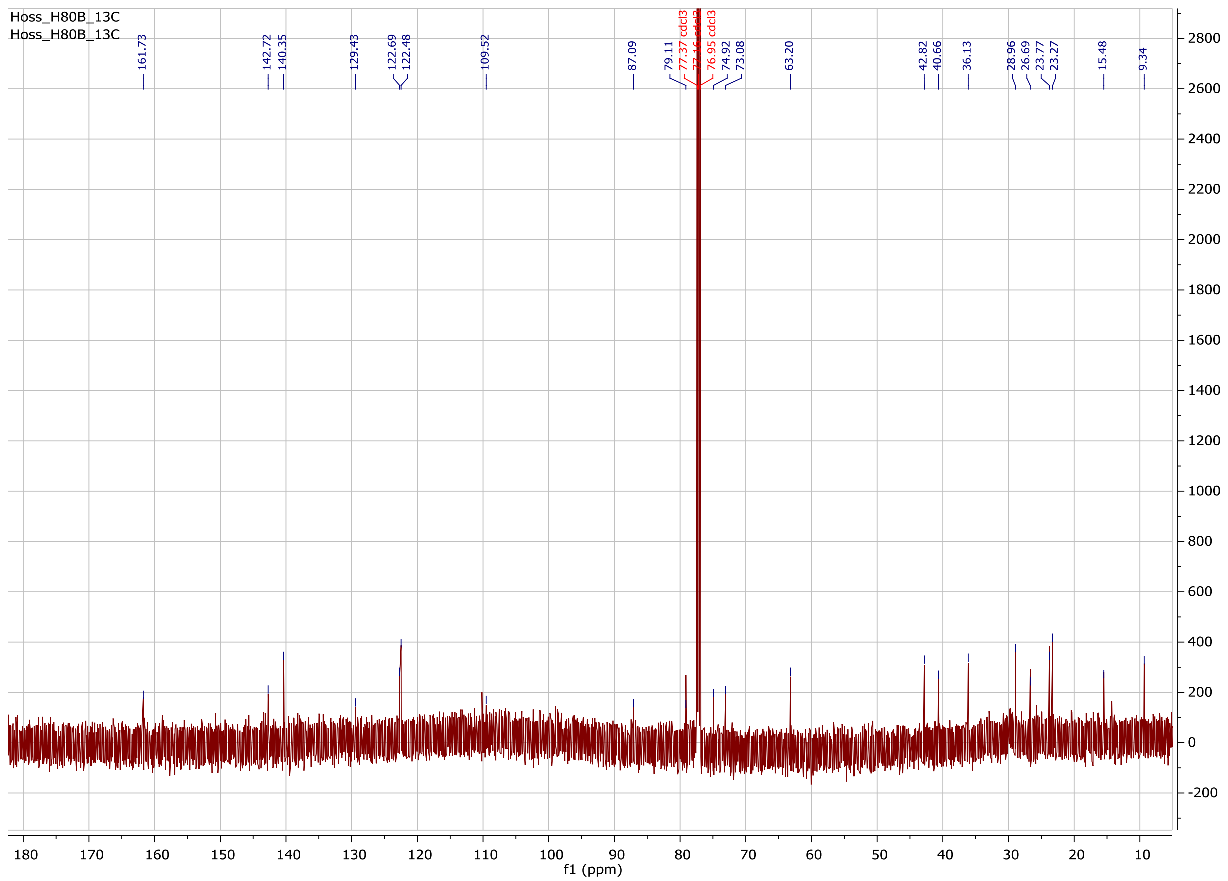

Fig.S25.The ^{13}C NMR (150 MHz, $CDCl_3$) spectrum of **4**

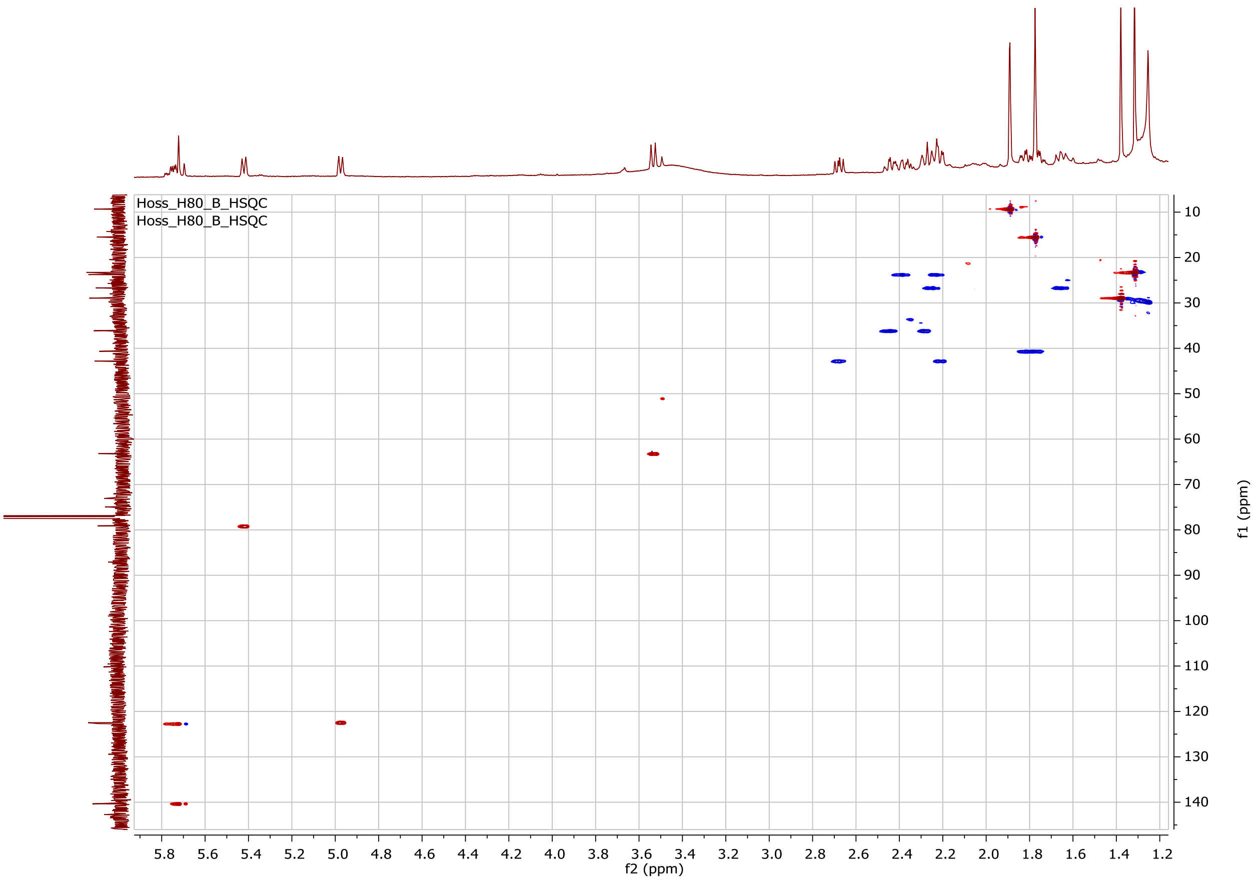

Fig.S26.The HSQC (600 MHz, $CDCl_3$) spectrum of **4**

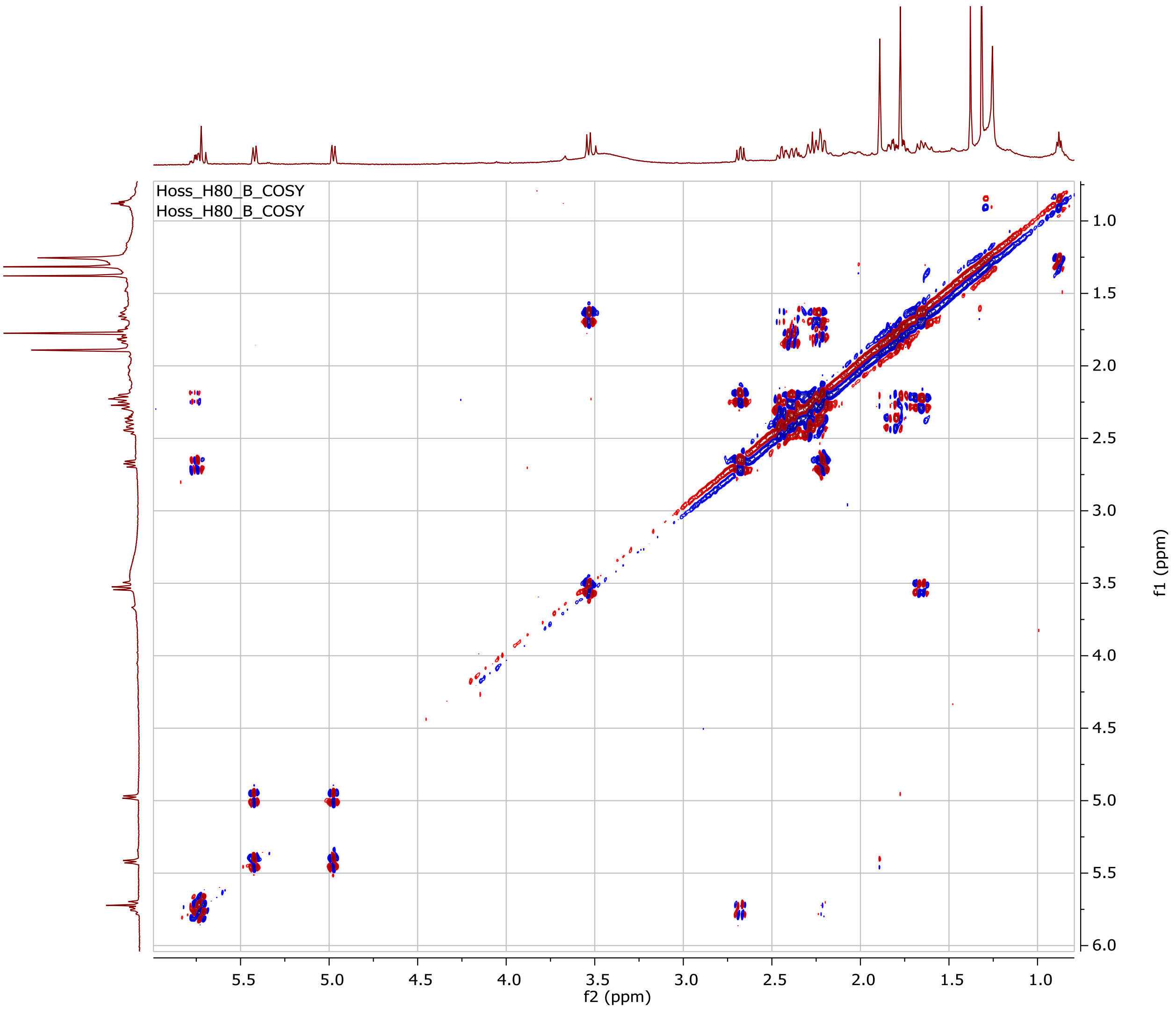

Fig.S27.The ^{1}H-^{1}H COSY (600 MHz, $CDCl_3$) spectrum of **4**

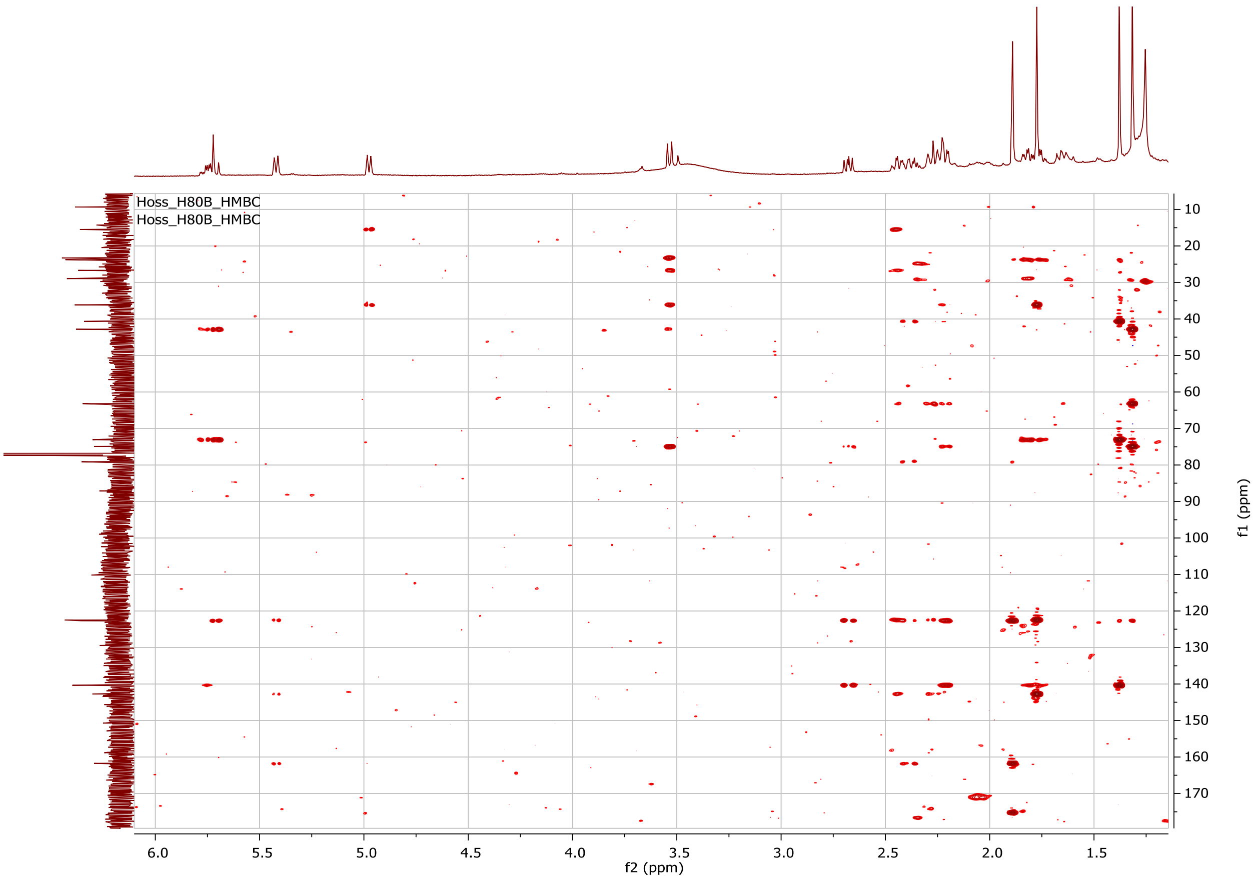

Fig.28.The HMBC (600 MHz, $CDCl_3$) spectrum of **4**

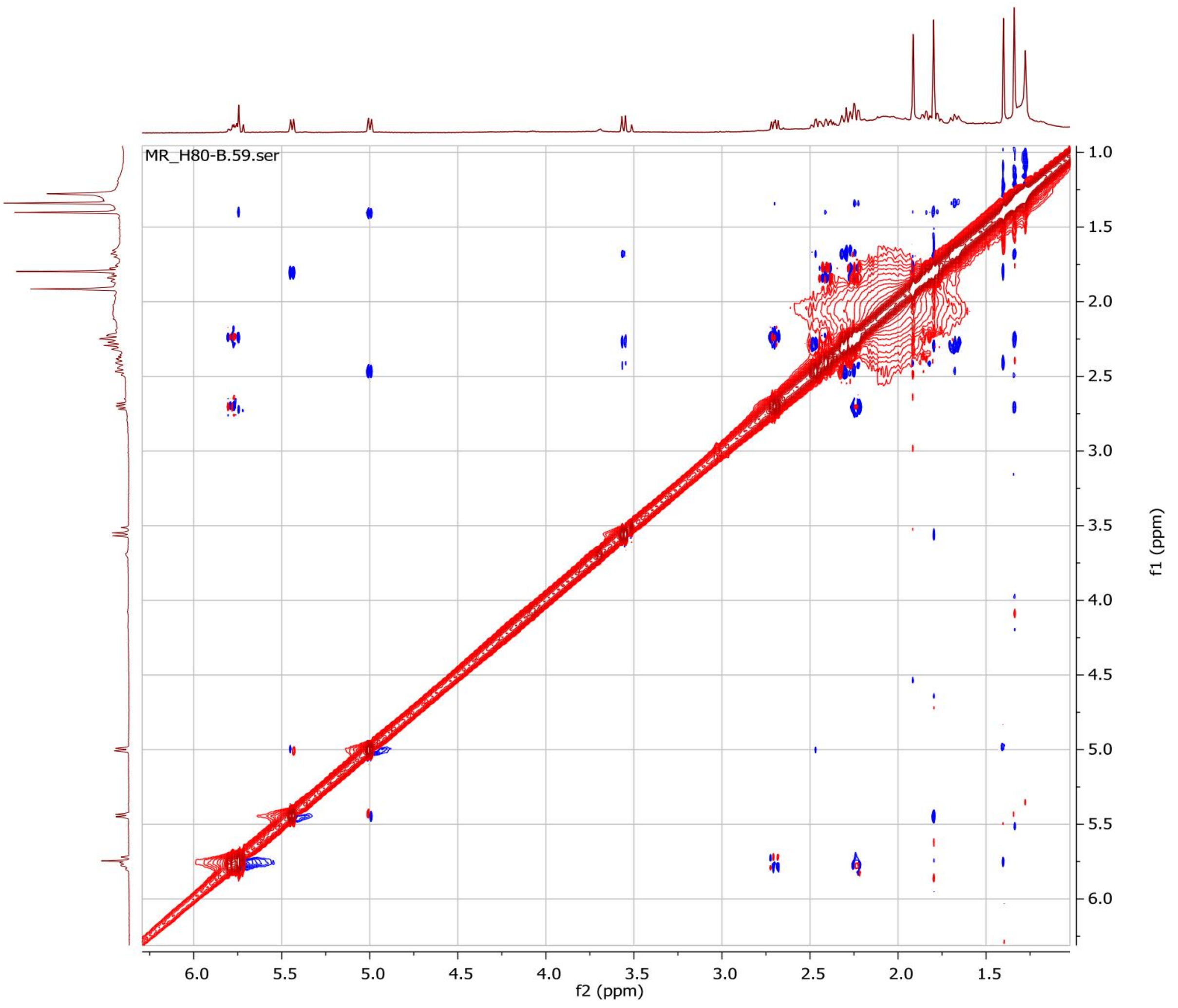

Fig.S29.The NOESY (600 MHz, $CDCl_3$) spectrum of **4**

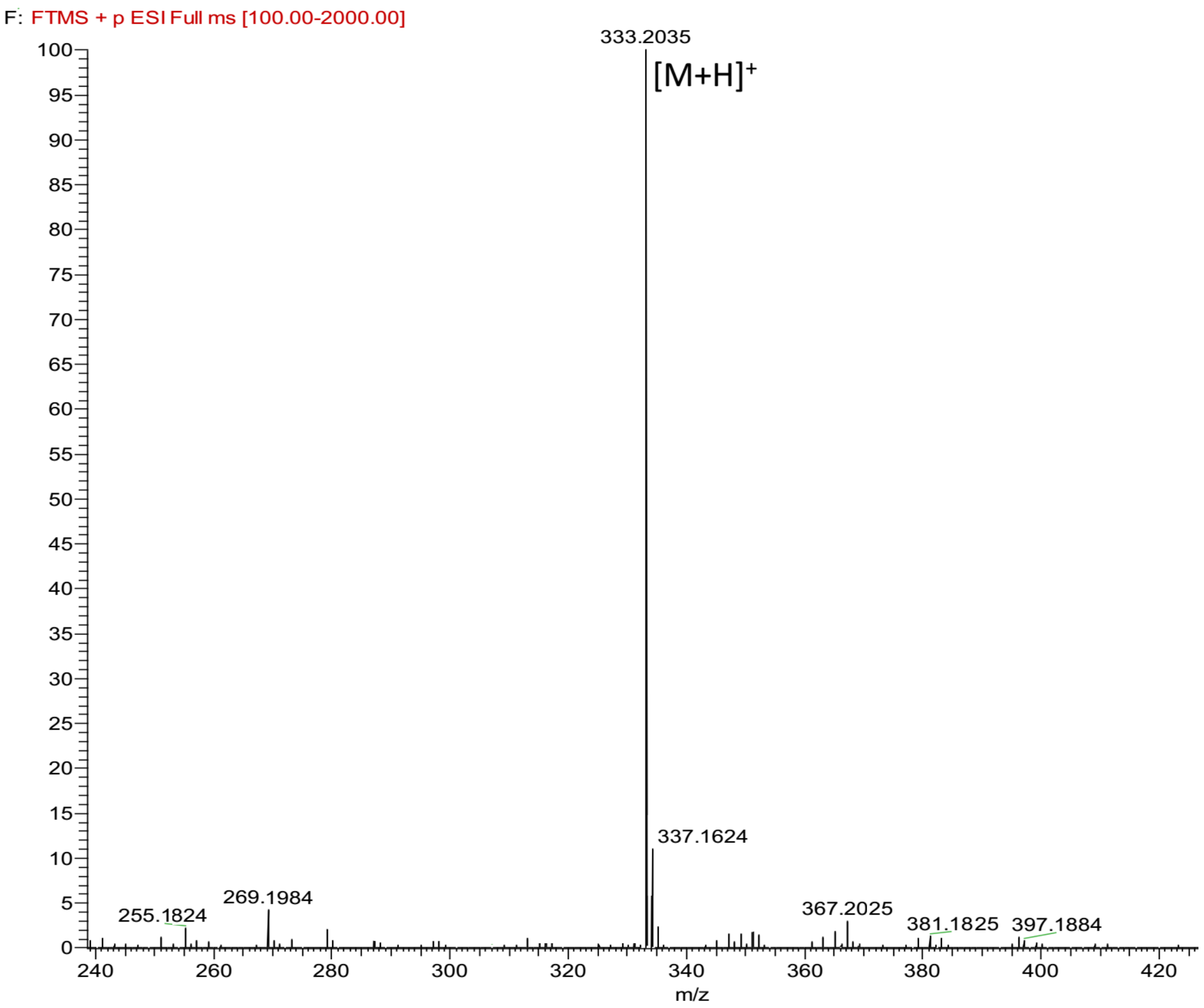

Fig.S30. The HRESIMS spectrum of **5**.

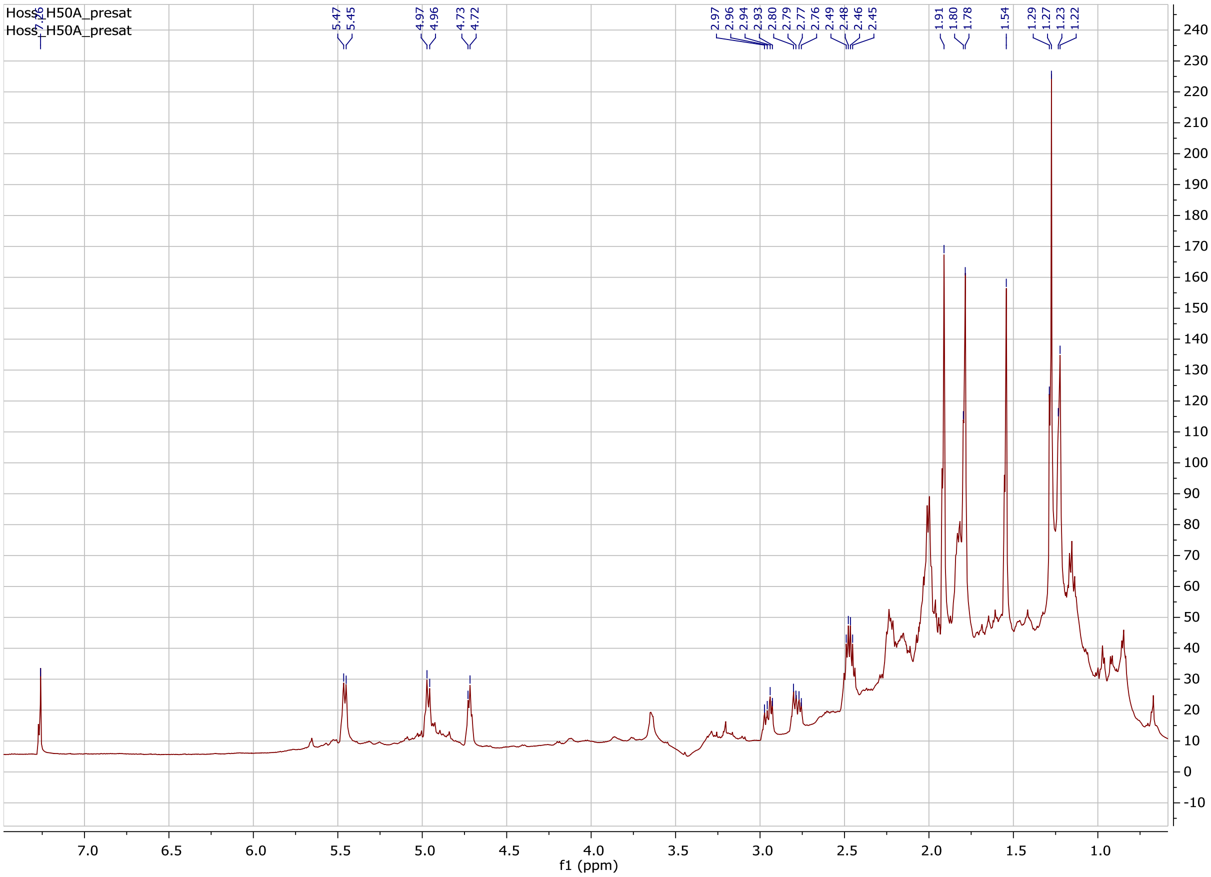

Fig.S31.The ^{1}H NMR (600 MHz, $CDCl_3$) spectrum of **5**

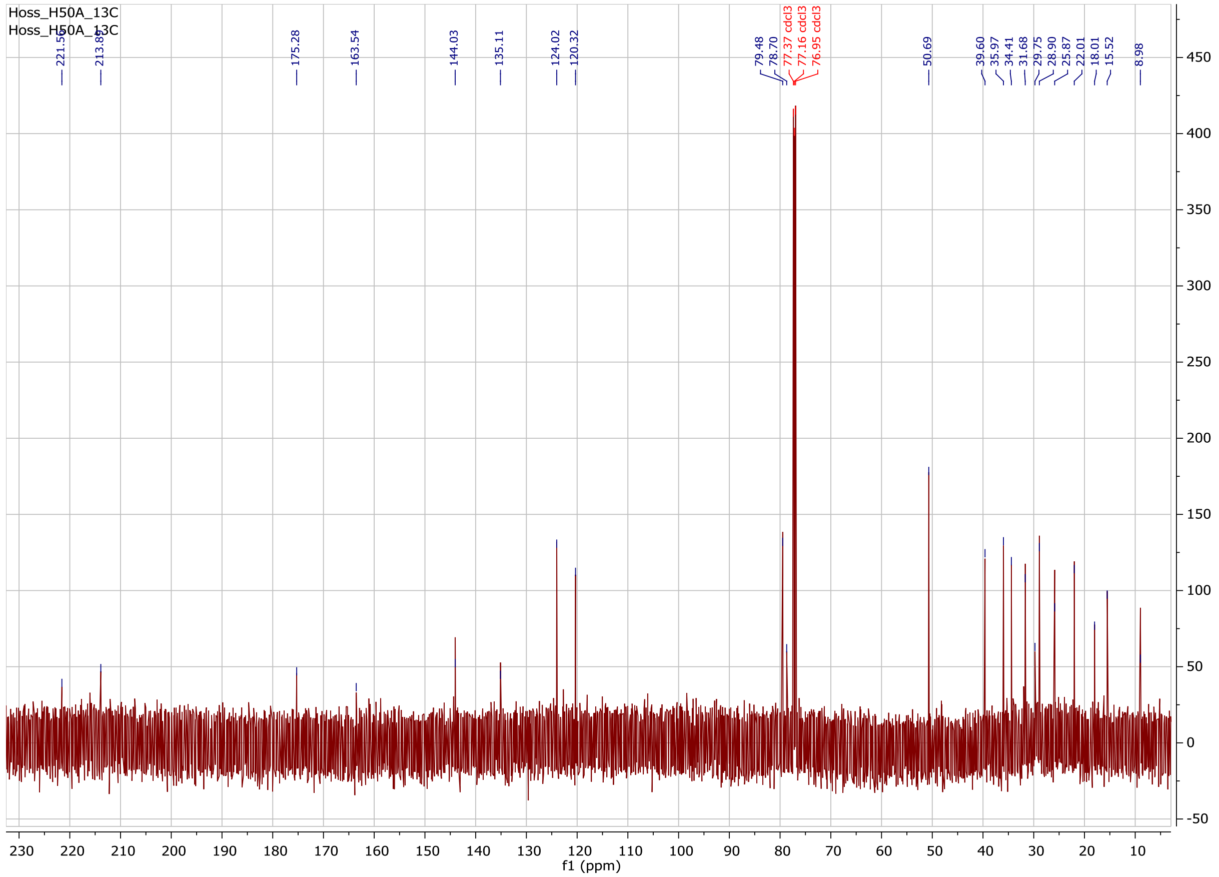

Fig.S32.The ^{13}C NMR (150 MHz, $CDCl_3$) spectrum of **5**

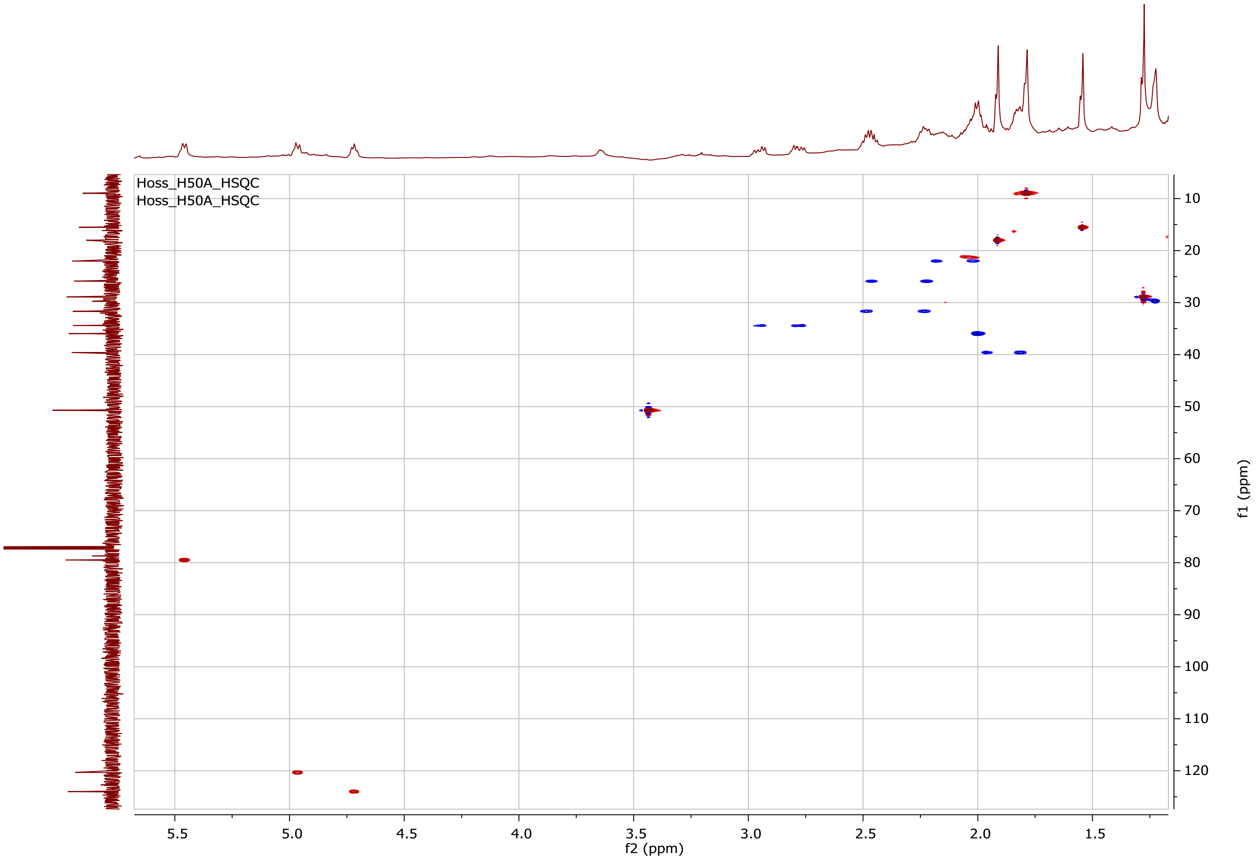

Fig.S33.The HSQC (600 MHz, $CDCl_3$) spectrum of **5**

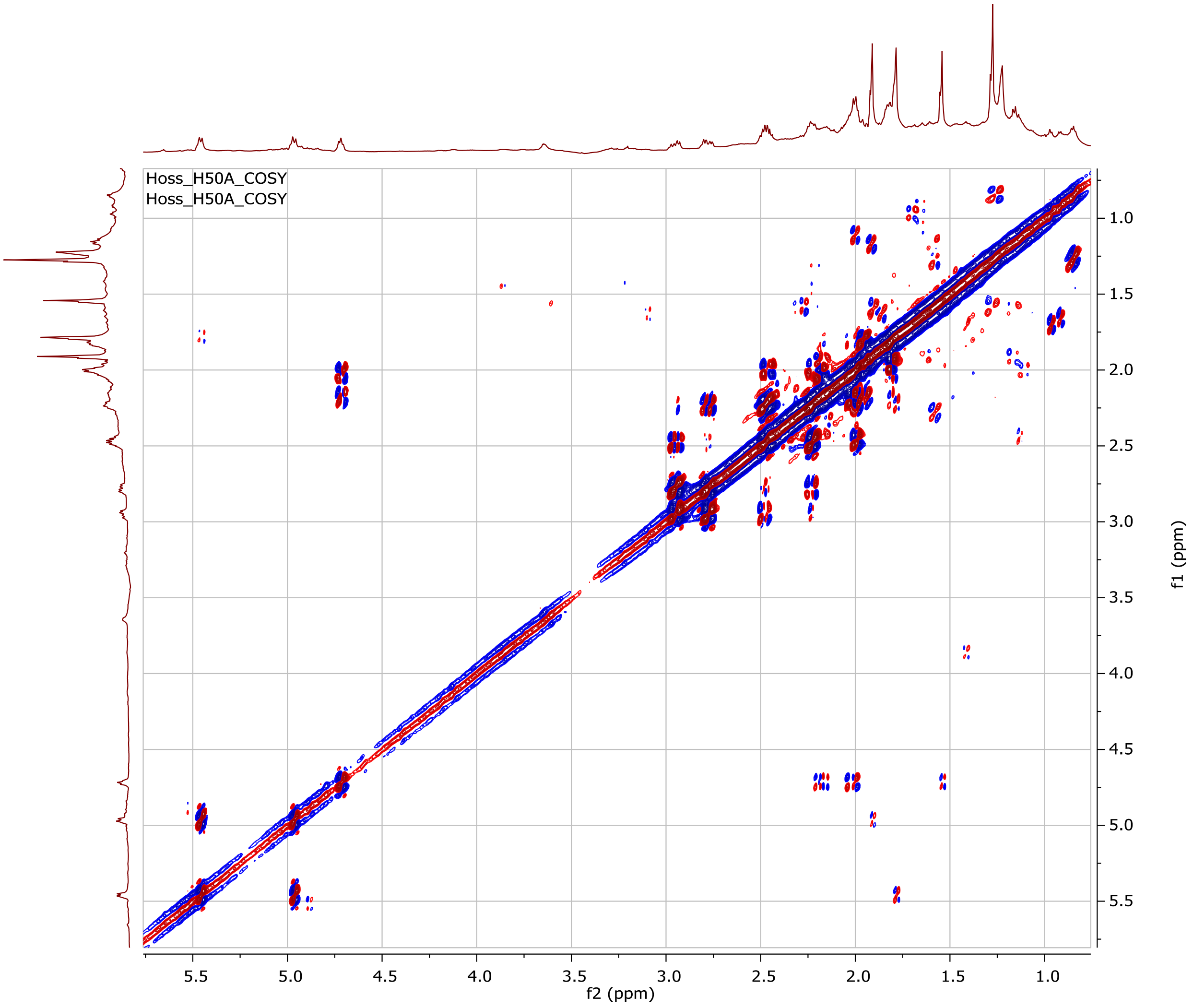

Fig.S34.The ^{1}H-^{1}H COSY (600 MHz, $CDCl_3$) spectrum of **5**

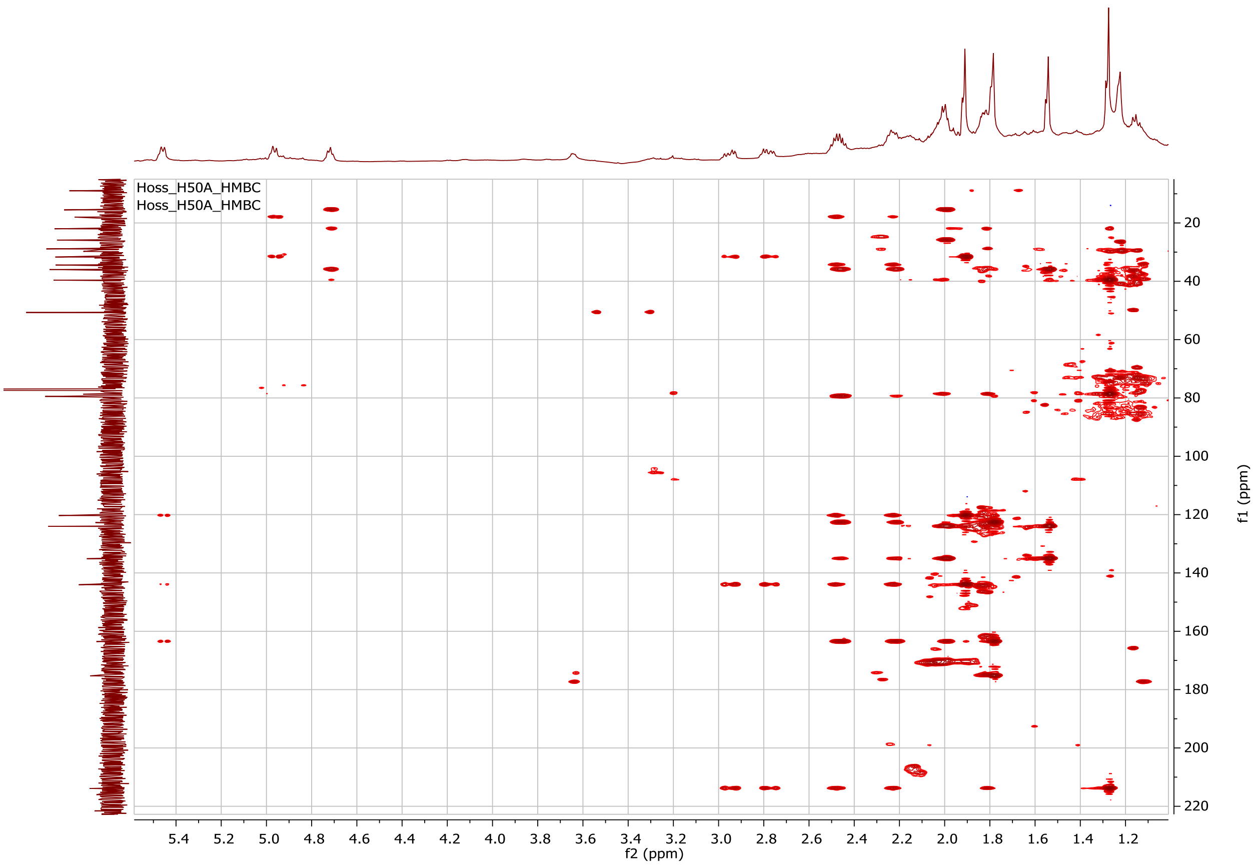

Fig.S35.The HMBC (600 MHz, $CDCl_3$) spectrum of **5**

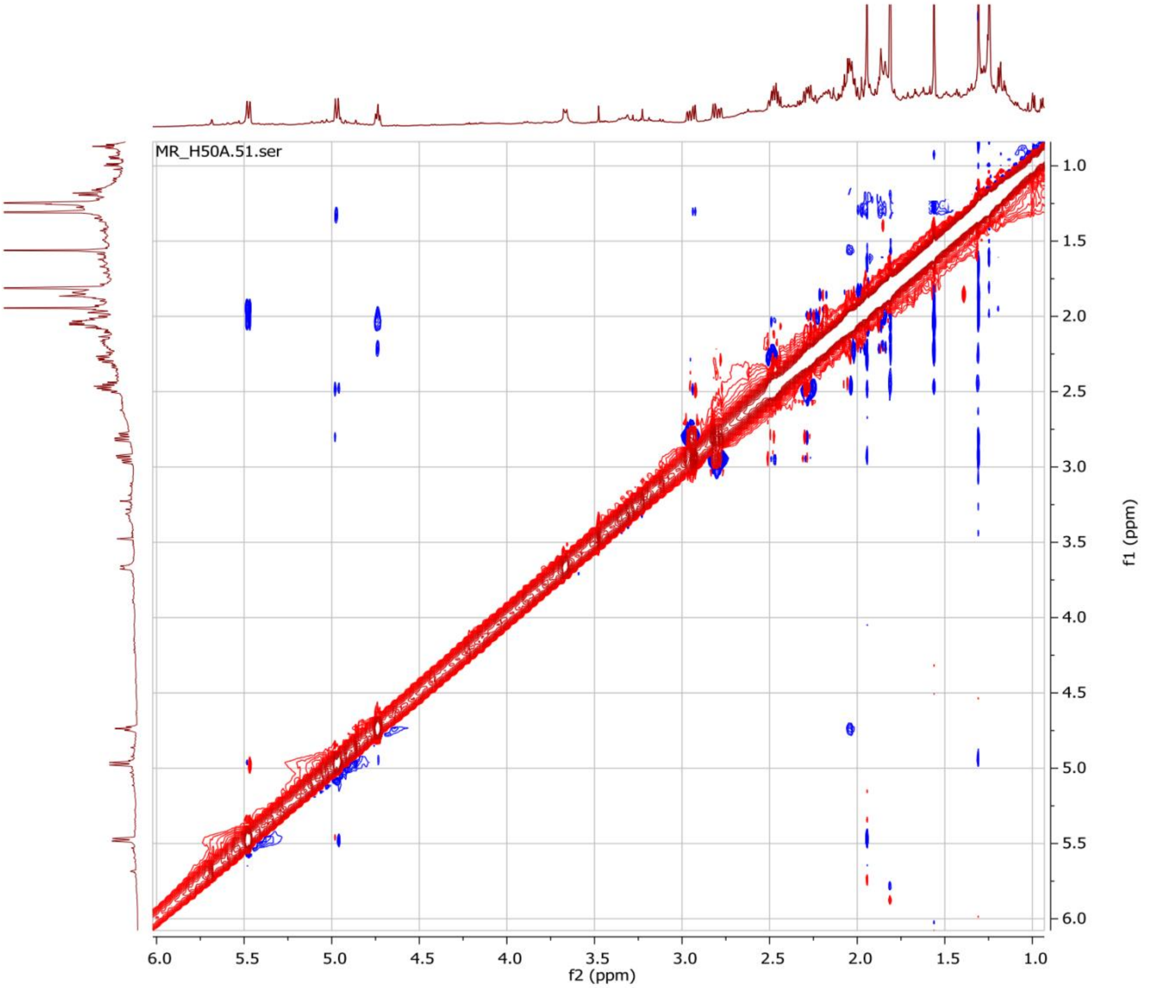

Fig.S36.The NOESY (600 MHz, $CDCl_3$) spectrum of **5**
